# Combined Impact of Irrigation, Potassium Fertilizer, and Thinning Treatments on Yield, Skin Separation, and Physicochemical Properties of Date Palm Fruits

**DOI:** 10.3390/plants12051003

**Published:** 2023-02-22

**Authors:** Hesham S. Ghazzawy, Nashi Alqahtani, Muhammad Munir, Naser S. Alghanim, Maged Mohammed

**Affiliations:** 1 Date Palm Research Center of Excellence, King Faisal University, Al-Ahsa 31982, Saudi Arabia; 2 Central Laboratory for Date Palm Research and Development, Agriculture Research Center, Giza 12511, Egypt; 3 Department of Food and Nutrition Sciences, College of Agricultural and Food Sciences, King Faisal University, Al-Ahsa 31982, Saudi Arabia; 4 Date Palm Research Center Al-Ahsa, Ministry of Environment, Water and Agriculture, Al Mubarraz 36321, Saudi Arabia; 5 Department of Agricultural and Biosystems Engineering, Faculty of Agriculture, Menoufia University, Shebin El Koum 32514, Egypt

**Keywords:** bubbler irrigation, machine vision, evapotranspiration, fruit quality, texture, color, skin puffiness

## Abstract

Orchard cultural practices, i.e., irrigation, fertilizer, and fruit thinning, are crucially encompassed to enhance fruit yield and quality. Appropriate irrigation and fertilizer inputs improve plant growth and fruit quality, but their overuse leads to the degradation of the ecosystem and water quality, and other biological concerns. Potassium fertilizer improves fruit sugar and flavor and accelerates fruit ripening. Bunch thinning also significantly reduces the crop burden and improves the physicochemical characteristics of the fruit. Therefore, the present study aims to appraise the combined impact of irrigation, sulfate of potash (SOP) fertilizer, and fruit bunch thinning practices on fruit yield and quality of date palm cv. Sukary under the agro-climatic condition of the Al-Qassim (Buraydah) region, Kingdom of Saudi Arabia. To achieve these objectives, four irrigation levels (80, 100, 120, and 140% of crop evapotranspiration (ETc), three SOP fertilizer doses (2.5, 5, and 7.5 kg palm^−1^), and three fruit bunch thinning levels (8, 10, and 12 bunches palm^−1^) were applied. The effects of these factors were determined on fruit bunch traits, physicochemical fruit characteristics, fruit texture profile, fruit color parameters, fruit skin separation disorder, fruit grading, and yield attributes. The findings of the present study showed that the lowest (80% ETc) and highest (140% ETc) irrigation water levels, lowest SOP fertilizer dose (2.5 kg palm^−1^), and retaining the highest number of fruit bunch per tree (12 bunches) had a negative effect on most yield and quality attributes of date palm cv. Sukary. However, maintaining the date palm water requirement at 100 and 120% ETc, applying SOP fertilizer doses at 5 and 7.5 kg palm^−1^, and retaining 8–10 fruit bunches per palm had significantly positive effects on the fruit yield and quality characteristics. Therefore, it is concluded that applying 100% ETc irrigation water combined with a 5 kg palm^−1^ SOP fertilizer dose and maintaining 8–10 fruit bunches per palm is more equitable than other treatment combinations.

## 1. Introduction

*Phoenix dactylifera* L., the date palm, has played a significant role in agricultural and socio-economic development throughout human history. Including the Kingdom of Saudi Arabia (KSA), it is a major crop in dry regions of North Africa and the Middle East, which provides food, nutrition, and building materials to the inhabitants [1,2]. There are around 9.61 million tons of dates produced worldwide from 1.25 million hectares of land [3]. In the KSA, the main fruits grown include date palm, olive, grape, citrus, mango, and guava. The date palm, among them, contributes 57% to the total fruit production and is grown in 63% of fruit-growing regions [4,5]. The KSA has a wide range of date palm diversity, with about 400 cultivated varieties, and is rated fourth in the world, with 1.54 million tons of production from 152 thousand hectares of area [3,6].

Water resources are limited in the date palm farming regions [7,8]. Water is also applied to the crops inefficiently, which depletes the available groundwater in the water scarce regions [9,10]. Although the date palm is relatively naturally adapted to withstand drought conditions in arid and semi-arid regions with high evapotranspiration and low rainfall conditions [11], any adverse changes in the soil environment directly or indirectly affect its growth and production [12,13]. In order to maintain optimum plant growth, it is therefore essential to supplement soil moisture using a variety of techniques, irrigation being the most important one. The method of irrigation system employed in water-scarce areas is crucial in determining the long-term sustainability of agriculture [14,15]. Globally, irrigation practices for food production use about 70% of freshwater [3]. However, the crops only use less than 60% of the irrigation water effectively [16]. Among the many strategies for water conservation, the adoption of a suitable irrigation system provides a feasible solution for inefficient water application [12,13,17,18]. In date palm orchards, surface irrigation techniques such as flood irrigation, furrow/basin irrigation, bubbler irrigation, drip irrigation, sprinkler irrigation, etc. are widely used. In many areas of the world including KSA where water is scarce, flood irrigation is still practiced, without considering the crops’ specific requirements, wasting significant amounts of water [19]. Date palm growers have also adopted the bubbler irrigation system to conserve water, which is largely squandered through flood irrigation and requires minimum filtration and maintenance [2,20,21]. Although it is claimed that the other irrigation systems—surface drip, sprinkler, trickle, and sub-surface drip—conserve more water than the bubbler system, many illiterate and low income farmers are unable to afford these sophisticated and expensive systems, which require specific technical knowledge and maintenance [22,23,24]. Studies have been conducted in various regions of Saudi Arabia to determine how much water the date palm requires. One study recommended between 7298.9 and 9495.2 m^3^ ha^−1^ of water for every 100 palm ha^−1^ [25], whereas other studies suggested 7300 m^3^ ha^−1^ [26], which indicates a variation within the regions.

Plants require nitrogen, phosphorus, potassium, calcium, magnesium, sulfur, iron, manganese, zinc, and other trace elements for their growth and development, which can be provided through organic and inorganic sources. Plant nutrients from organic sources decompose slowly and take longer for plants to absorb, whereas they are readily available in inorganic chemical fertilizers [27,28]. Mineral fertilizers or combined organic-inorganic fertilizers have been shown to have a positive impact on food production globally and are an essential part of many agricultural production systems [29]. Unfortunately, the general belief among date palm growers is that they can grow and produce fruits successfully without adding organic-inorganic fertilizers; however, research studies have contradicted this [30,31,32]. The use of fertilization management, based on the application of both compost and nitrogen, could improve the yield of date palms and increase farmers’ income while also restoring soil fertility [33]. The fruit quality of cvs. Khalas and Khassab was greatly improved by the use of NPK fertilizer along with micronutrients and organic peat [34]. The growth of juvenile date palm plants was also enhanced by the use of organic materials such as chitosan, organic fertilizer, amino acids, and seaweed extract [35]. The number of leaves and bunches per palm, fruit yield, total sugar content, fruit quality, and mineral content of the leaves were all improved in cv. Sewy when a higher dose of NPK fertilizer was applied; however, the biannual bearing was decreased [36]. Similarly, the dry matter of young date palm seedlings was significantly enhanced when poultry manure was applied with N and K fertilizers [37]. The fruit yield and quality of cv. Dhakki was enhanced by the application of nitrogen alone or in combination with phosphorus and potassium fertilizers [30,31]. In Valencia orange, the application of potassium reduced fruit splitting, increased fruit size, peel thickness, and fruit acidity [38].

Fruit thinning practices, whether manual, mechanical, or chemical, largely alter crop load, which has an impact on fruit yield and quality. Fruit size and thinning intensity are closely associated. Along with crop load, other factors influencing the response to thinning includes fruit bud quality and their competition within the bunch [39,40]. It is an important cultural practice to improve the physicochemical properties of fruit such as fruit retention, bunch weight, fruit weight, fruit length and diameter, and sugar content. In addition, it also reduces the alternate fruit bearing of some date palm cultivars [17,41]. Thinning by removing 30% bunch per palm, or fruit thinning by either cutting back 30% of strand tips or removing 30% of a total number of strands from the center of a bunch significantly increased fruit size, total soluble solids, and sugar contents. In contrast, total acidity decreased considerably in date palm cv. Zaghloul [42]. Maximum fruit size, weight, total soluble solids, yield per palm, minimum stone pulp ratio, and fruit drop were measured by 50% strand thinning in cv. Kur [43]. Strand thinning (30%) after four weeks of pollination significantly improved the fruit quality of date palm cv. Succary [44].

The fruit’s skin supports its structure and protects it from external biotic and abiotic stressors [45]. The skin can sometimes split or crack from the flesh as it ripens [46]. It could affect fruit growers’ potential to market their produce commercially. Fruit skin separation is a negative commercial characteristic worldwide in various fruits. Skin-separated fruits are not exported [47] and their price is only half of fruits without skin separation [48,49]. The phenomenon does not occur in some cultivars (cvs.) of date palm, whereas it does in others. It is mostly reported in cvs. Khalas, Sukary, Sheshi, Hillawi, Khizrawi, Khanizi, Zahidi, Sagai, Barhee and Majhul, which have a great economic importance [48,50,51]. Various factors are responsible for that undesirable fruit trait, such as genetic factor, pollinator source, soil and water factors, environmental factors (temperature, wind, relative humidity), and fruit thinning [51,52]. The mechanical characteristics of the cuticle and skin cell walls may have an impact on how easily fleshy fruits split [52]. The author of [48] compared the two date palms cvs. Dayri, where skin separation does not occur, and Barhee, whose fruits strongly produce skin separation, and reported that both cultivars have different skin characteristics. The skin of cv. Barhee fruits is less flexible and more rigid, resisting artificial stretching force, and tends to form folders while detaching from inner soft tissue, whereas cv. Dayri fruits have the opposite characteristics. The epidermis cells’ line order in Barhee and circular order in Dayri are two factors contributing to this feature. Deficit irrigation at 55% ETc significantly reduced the skin cracking and separation in fig [53]. It has been suggested that the date palm cv. Madjool’s early fruit ripening can minimize skin separation disorder. By implementing good cultural practices, such as avoiding excessive irrigation and nitrogen fertilizer, the delay in ripening can be mitigated [54]. An anatomical study in cv. Mejhoul indicated that sclereid cells were abundant in skin-separated fruits compared to normal ones, at the Tamr stage. These cells formed a chain of aggregates and were closer to the cuticle, leading to the partial separation of the exocarp from the fruit mesocarp [55].

Date palm is the major staple fruit tree crop in the KSA. Most of the country’s area comprises of desert sandy soils, where water and fertilizer retain poorly. Management practices such as irrigation and fertilization play a key role in the fruit yield, quality, and skin separation incidence, particularly when combined with cultural practices such as fruit thinning, which increases fruit size and quality and modifies the fruit’s osmotic and turgor potentials. The impact of these variables is often ignored by low income date palm farmers and entrepreneurs involved in the date palm industry who are mainly uneducated, unaware of scientific and technical knowledge, and untrained. They usually over- or under-irrigate date palm orchards, ignore the worth of fertilizer application, and avoid bunch load management to reduce the labor cost. Many previously mentioned studies have shown the significant effects of these variables on date palm yield and quality. To our knowledge, no one has studied the combined impact of these three key orchard cultural practice variables on date palm fruit grading, texture, yield, and quality of date palm cv. Sukary. A couple of studies have been conducted on date palm skin separation disorder, which were based on their mechanical properties [48] and anatomical traits [55]. However, in the present study, the combined effect of these three key variables was also focused on the fruit texture profile, skin separation disorder, and fruit grading of date palm cv. Sukary. The outcome of the present study provides guidance to the growers involved in the date palm industry for suitable irrigation scheduling, fertilization, and bunch thinning management decisions.

## 2. Results and Discussion

### 2.1. Applied Irrigation Water

**Figure 1:**
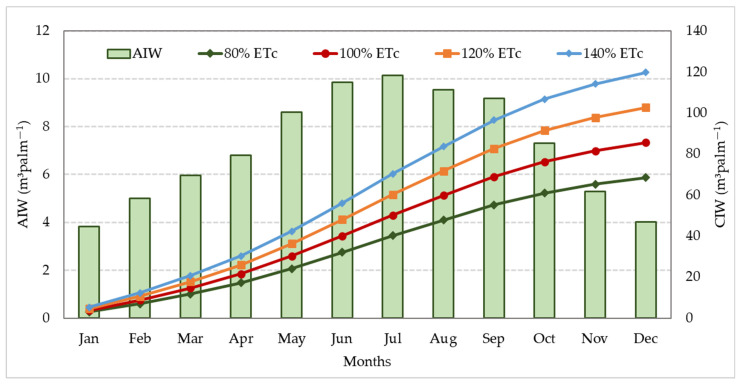
Average monthly applied irrigation water (AIW) at 100% ETc and the cumulative irrigation water (CIW) applied at four irrigation water levels (80, 100, 120, and 140% ETc) during seasons 2020/2021 and 2021/2022.

Figure 1 shows the average monthly applied irrigation water amounts at 100% ETc, and the average cumulative water per date palm tree over the two years studied, under the four irrigation water levels (80, 100, 120, and 140% ETc). The ETo was determined using the Penman–Monteith equation. The ETc was calculated by multiplying the ETo by the crop factor (kc = 0.95). The average monthly applied irrigation water amount was calculated by multiplying the ETc with the irrigation area of the date palm trees (31.15 m^2^). The average values of the actual applied irrigation water per date palm tree did not differ across the assessed irrigation replicates. During the seasons 2020/2021 and 2021/2022, the average cumulative irrigation water under the different irrigation water levels (80, 100, 120, and 140% ETc) was 68.46, 85.57, 102.69, and 119.80 m^3^ palm^−1^, respectively.

### 2.2. Fruit Bunch Characteristics

Considering the interaction between the three factors, the findings showed the significant (*p < 0.05*) interaction effects of several combinations of different irrigation levels, SOP fertilizer doses, and bunch thinning treatments on bunch length, stalk width, empty bunch weight, and strand length (Table 1). Data indicated that the maximum bunch length, stalk width, empty bunch weight, and strand length were observed when 100% ETc irrigation water was applied, followed by 120% ETc irrigation water. Similarly, the application of the 5 kg palm^−1^ SOP fertilizer dose significantly improved these parameters, followed by the 7.5 kg palm^−1^ SOP fertilizer dose. The highest bunch length, stalk width, empty bunch weight, and strand length were observed when there were only 8 fruit bunches per palm, followed by 10 fruit bunches per palm. Overall, irrigation at 100% ETc + 5 kg palm^−1^ SOP fertilizer dose, and 8 fruit bunches thinning treatment gave the highest bunch length, stalk width, empty bunch weight, and strand length. The highest bunch weight was recorded when cv. Mazafati was irrigated at 70% ETc at intervals of 100 mm evaporation [56]. On the other hand, removing 20 or 30% thinning of bunch number per palm significantly increased the bunch weight in cv. Zaghloul [42]. Similarly, the foliar application of potassium citrate enhanced the bunch weight in cv. Hayani [57]. In cv. Seweda, the application of SOP, 2 kg palm^−1^ soil broadcast along with 2 kg palm^−1^ foliar spray significantly enhanced the bunch weight in the second year of study [58].

### 2.3. Physicochemical Characteristics of Fruit

Data regarding fruit length, fruit width, fruit weight, seed weight, pulp weight, and seed pulp ratio were significantly (*p <* 0.05) affected by different irrigation levels, SOP fertilizer doses, and bunch thinning treatment (Table 2). Maximum fruit length, fruit width, and fruit and pulp weight were recorded at 100% and 120% ETc levels, 5 and 7.5 kg palm^−1^ SOP fertilizer application, and 8 and 10 bunches palm^−1^ treatment combinations, which was followed by the 140% ETc level and 7.5 kg palm^−1^ SOP fertilizer treatment combination. These parameters were significantly minimum at the 80% ETc level regardless of any SOP fertilizer and bunch thinning treatments. However, the seed weight and seed pulp ratio were higher at the lowest irrigation level (at 80% ETc) at all SOP fertilizer (2.5, 5, and 7.5 kg palm^−1^) and bunch thinning (8, 10, and 12 bunches palm^−1^) treatments. Palm trees irrigated at 100% and 120% ETc levels, 5 and 7.5 kg palm^−1^ SOP fertilizer doses, and retaining 8 and 10 bunches palm^−1^ had the lowest seed weight and seed pulp ratio.

A significant (*p <* 0.05) interaction effect was observed between irrigation levels, SOP fertilizer doses, and thinning treatments on fruit TSS, titratable acidity, TSS and titratable acidity ratio, total sugars, and moisture contents; however, there was a non-significant effect of these factors on the fruit water activity variable (Table 3). As indicated in the table, the highest TSS was determined at 100% ETc combined with the 5 kg palm^−1^ SOP fertilizer application, which was closely followed by 120% ETc at the same SOP fertilizer dose. The table indicating the thinning treatments showed that the TSS was higher when the number of fruit bunches per palm retained 8–10 per palm. The titratable acidity was higher when date palm trees were irrigated at 100% ETc, irrespective of any SOP fertilizer doses and bunch thinning levels, which was followed by 120% ETc. The lowest titratable acidity was noted at 80% and 140% ETc levels at all SOP fertilizer doses and bunch thinning treatments. Table 3 also depicts that the maximum TSS and titratable acidity ratios were recorded at 140% and 80% ETc levels at all SOP fertilizer doses and bunch thinning treatments, which was significantly reduced at 100% and 120% ETc irrigation levels. Data regarding the total sugar content showed that it was maximum when the date palm trees were irrigated at 100% and 120% ETc levels and received the 5 kg palm^−1^ SOP fertilizer dose. The higher dose of SOP fertilizer (7.5 kg palm^−1^) was also statistically at par when it was combined with the 100% ETc irrigation level. Although the bunch thinning treatment also significantly differed when compared with other factor’s combinations, there was a statistically non-significant difference between the three thinning treatments when this factor was combined with the 100% ETc level and 5 and 7.5 kg palm^−1^ SOP fertilizer doses, and the 120% ETc level and 5 kg palm^−1^ SOP fertilizer dose. Fruit moisture content was increased when the irrigation water and SOP fertilizer was increased. It was higher in fruits harvested from trees irrigated with the highest level of water (at 140% ETc) and applied highest quantity of SOP fertilizer (7.5 kg palm^−1^), followed by irrigation water applied at 120% and 100% ETc levels, combined with the highest SOP fertilizer dose (7.5 kg palm^−1^). The response of the bunch thinning levels was more or less similar when combined with the highest irrigation and SOP fertilizer factors. However, the fruit moisture content was highest when the number of bunches was maintained from 8 to 10 per palm. Despite being statistically non-significant, the water activity parameter increased linearly with the increase in irrigation water and ranged between 0.419–0.457.

Since the date palm fruit is becoming more popular, both date growers and consumers are interested in the demand for high quality date palm fruit. Irrigation, fertilizer management, and fruit thinning play a vital role in the enhancement of fruit quality. Our findings showed that the fruit quality-related attributes (fruit size, fruit and pulp weight, TSS, titratable acidity, and total sugars) were improved at the optimum application of irrigation water, potassium fertilizer, and bunch thinning treatments. These results could be explained by the efficient water and nutrient use of the functional absorbing root zone. Plants grown in low water conditions accumulated more sugars and had higher solute concentrations, which led to higher total soluble solids [59,60]. Date palm cv. Khalas irrigated at 75% ETc had the highest fruit size, pulp weight, TSS, and total and reducing sugars [61]. Deficient (50% ETc) or excessive (150% ETc) irrigation water applied to cv. Succary had a negative effect on fruit quality [62]. Potassium sulphate (4.5 kg palm^−1^) applied to cv. Bartamoda significantly enhanced the fruit size, weight, TSS, sugars, and mineral contents [63]. Fruit quality attributes were also improved by the application of potassium sulphate in cvs. Hayany [64] and Barhee [65]. In cvs. Zaghloul and Haiany, the removal of 20–25 strands per bunch significantly improved their physicochemical characteristics [66,67]. Similarly, 30% strand thinning had a significantly beneficial effect on the fruit quality of cv. Succary [44]. In the present study, the fruit water activity was well below the critical level in all treatment combinations that facilitate microorganism growth. When water activity is lower than 0.90–0.95, many bacterial species cannot grow. Likewise, toxin production is also inhibited when the water activity is lower than this value. Mold and yeast cannot thrive in environments with water activity levels between 0.80 and 0.88. However, some osmophilic yeasts can still grow on substrates with water activities as low as ~0.60 [68,69]. Muhammad et al. determined a water activity of 0.56 in the stored date palm of cv. Sukary [70]. There was a non-significant effect of deficit irrigation treatments on water activity of almond [71].

### 2.4. Fruit Texture Profile Analysis

**Figure 2:**
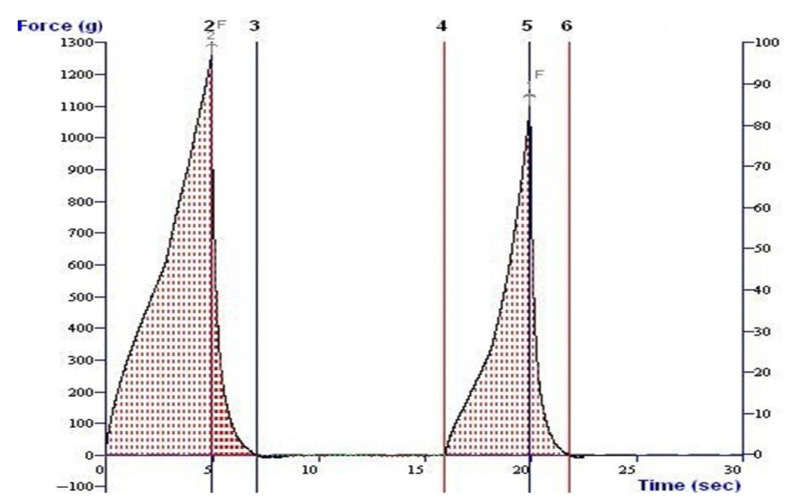
Typical Force-Time curve from one of the samples for texture profile analysis of date palm cv. Sukary.

One of the essential date quality factors is fruit texture, and the texture profile analysis provides insightful information regarding fruit quality. Therefore, texture profile analysis was performed for the date palm cv. Sukary fruits obtained from trees subjected to different irrigation levels, SOP fertilizer doses, and bunch thinning treatments, and the results were represented in Table 4. The texture profile analysis curve (Figure 2) was taken into account when calculating all textural characteristics such as hardness, springiness, cohesiveness, gumminess, chewiness, and resilience. In terms of date palm fruit hardness, springiness, cohesiveness, gumminess, chewiness, and resilience, a significant (*p <* 0.05) interaction impact was observed between irrigation levels, SOP fertilizer doses, and thinning treatments (Table 4).

The ripening of date palm fruit is mostly determined by the fruit’s firmness. Fruits that have started to ripen typically have softer fruit hardness than they did before. The peak force during the first compression cycle is what is known as the hardness, which is a crucial textural metric connected to the strength of the fruit structure under compression [72]. The findings of the present study indicated that the highest fruit hardness was determined when lowest level of irrigation water was applied (at 80% ETc), irrespective of SOP fertilizer and fruit thinning treatments. Fruit hardness decreased with the increase in irrigation water. Fruit hardness was reduced with the combination of higher irrigation water (100%, 120%, and 140% ETc) with the 5 and 7.5 kg palm^−1^ SOP fertilizer doses. Fruits were harder when date palm trees received the lowest dose of SOP fertilizer application (2.5 kg palm^−1^). Retaining 8–10 fruit bunches per palm was mostly at par with, and produced comparatively soft fruits to the 12 bunches per palm treatment. A mechanical textural property known as springiness relates to the rapidity and degree of recovery from a deforming force. The results showed that the highest fruit springiness was measured at the 100% ETc irrigation level, when 5 kg palm^−1^ SOP fertilizer was applied and the number of fruit bunches was maintained at 8−10 palm^−1^. A food’s degree of deformability before it breaks is referred to as its cohesiveness, which is a mechanical textural characteristic. Fruit cohesiveness was higher when the minimum level of irrigation water was applied (at 80% ETc) at all SOP fertilizer and thinning treatments. However, it was minimum when date palm trees received the 5 kg palm^−1^ SOP fertilizer dose at 100%, 120%, and 140% ETc levels. Palms retaining 8 and 10 bunches palm^−1^ also showed encouraging results when combined with the 5 kg palm^−1^ SOP fertilizer treatment.

The fruit gumminess was higher at low irrigation levels at all SOP fertilizer and thinning treatment combinations; however, it was decreased at the 80% ETc level combined with the 5 and 7.5 kg palm^−1^ SOP fertilizer doses. Irrigation water applied at the 120% ETc level in combination with 5 kg palm^−1^ SOP fertilizer was statistically at par. The amount of energy required to masticate solid food is known as chewiness [72]. It was higher in the harder of the date palm fruits, which were produced when trees were subjected to the lowest irrigation water (at 80% ETc). However, it was recorded at its minimum in trees irrigated at the 100% ETc level, followed by the 120% ETc level. SOP fertilizer treatment applied at 5 kg palm^−1^ also indicated the lowest chewiness compared to other SOP doses. Data regarding bunch thinning indicated that fruits from the 8−10 bunches palm^−1^ treatments had the lowest chewiness. The ability of a sample to recover during an initial compression is referred to as its resilience [73]. Table 4 showed that the highest fruit resilience values were measured at the 100% ETc level and when 5 and 7.5 kg palm^−1^ SOP fertilizer was applied, followed by the 120% ETc level and 5 kg palm^−1^ SOP fertilizer treatment combination. Fruit thinning levels showed more or less similar results when combined as a factor with the aforementioned irrigation and SOP fertilizer treatments.

There are no earlier research reports on how irrigation, fertilizer, and thinning practices affect the texture parameters of date palm fruit. The fruit of the date palm should not be too hard or too soft in order to maintain the desired textural qualities. Date palm fruit texture varies among the cultivars; for example, the fruit hardness of cv. Reziz is higher than that of cvs. Sheshi and Khalas, while fruit springiness, cohesiveness, and resilience is higher in cv. Sheshi followed by cv. Khalas. Therefore, due to their exceptional texture qualities of springiness, cohesiveness, and resilience, date palms cvs. Sheshi and Khalas have a high consumer preference in Saudi Arabia [74]. In Saudi Arabia, the economic value of date palm fruits mostly depends on their texture, particularly the hardness, and soft or semi-dried cultivars are preferred by consumers and fetch more money [75]. Fruit texture is also influenced by the climatic condition of the growing region. The climatic conditions of Tunisia, Oman and the UAE favor semi-dry and dry date palm cultivars, while soft dates are predominantly grown in Bahrain, Kuwait, and Qatar [76]. Based on the texture profile analysis attributes, [22] categorized market dates into three groups: hard-resilient, soft-springy, and firm-adhesive. In pear, the fruit firmness increased with both a higher dose of potassium and the number of sprays [77]. Fruit firmness was significantly higher when olive trees were exposed to a water stress condition, compared to irrigated ones [78]. The reduction of irrigation water (50% ETc) also had a negative effect on the fruit firmness of blueberry [79]. A positive correlation between calcium spray and fruit firmness was found in blueberries [79] and apple [80]. Fruit firmness was higher at the application of 400 and 600 mg L^−1^ potassium in melons [81]. Fruit firmness is influenced by a variety of factors, including both positive and negative ones, such as harvest maturity, dry matter, mineral content, enzyme activity, fruit size, cell number and size, intercellular space, and specific gravity [82]. Fruit firmness at harvest can be affected by a wide variety of thinning-related factors. The heavy cropping caused by the reduced thinning practice may limit the amount of carbohydrates available for the formation of cell walls. Fruit firmness may be improved through thinning since it enhances the fruit’s dry matter content [40]. In this context, it is important to note that in the present study, the higher values of hardness, cohesiveness, gumminess, and chewiness, as well as the lower springiness and resilience values of dates produced by trees which received the minimum irrigation water (at 80% ETc) at all SOP fertilizer and bunch thinning treatment combinations, or the 140% ETc level combined with the 2.5 kg palm^−1^ SOP fertilizer dose, are not desirable textural properties. However, these textural qualities were ideal at 100% and 120% ETc levels combined with the 5 and 7.5 kg palm^−1^ SOP fertilizer doses and 8–10 fruit bunches thinning per palm treatments.

### 2.5. Fruit Color Characteristics

In addition to the texture profile analysis, color is also a crucial sensory and physical attribute that directly influences the quality of a product. It is a sensory perception and defined as the physiological response to a physical stimulus [83]. Color analysis was performed for all date palm fruit samples, and the values of color parameters (L, C, h° and ΔE) are shown in Table 5. It was found that there were significant (*p* < 0.05) differences in values of the color parameters. The data in Table 5 confirmed that the cv. Sukary date fruits were the lighter (L) when date palm trees received the lowest dose of SOP fertilizer (2.5 kg palm^−1^) at all irrigation levels (80%, 100, 120, and 140 ETc), and at all bunch thinning treatments. Whereas the lowest values of L were observed when the SOP fertilizer dose was increased to 5 kg palm^−1^ in almost all irrigation levels, particularly at 100% ETc, which indicated a darker color of the fruit. Data regarding chroma (C) color showed that fruit were brighter at the 80% ETc level combined with the 2.5 and 7.5 kg palm^−1^ SOP fertilizer doses, followed by the 100% ETc level combined with the 5 and 7.5 kg palm^−1^ SOP fertilizer doses. The higher hue angle (h°) was recorded at the 80% ETc level combined with the 2.5 kg palm^−1^ SOP fertilizer dose, whereas it was minimum at the 100% ETc level combined with the 5 kg palm^−1^ SOP fertilizer dose. The total color difference (∆E) was significantly higher in all irrigation levels combined with the 2.5 kg palm^−1^ SOP fertilizer dose, which was significantly lowest in the 120% ETc level + 5 kg palm^−1^ SOP fertilizer application. A more or less varied response was observed regarding the bunch thinning treatments for different color parameters.

The mature fruit of the date palm varies in color from deep red to pale yellow, depending on the cultivars; for example, the fruits of cvs. Khalas and Sheshi had the highest ∆E compared to cv. Reziz [74]. The fruit color also changes at different growth stages; specifically, the hababouk, kimri, khalal, biser, rutab, and tamr stages. It is measured by color index *a* and *b*, as a function of their anthocyanin level [84,85,86,87]. Anthocyanins are an important group of pigment-containing molecules produced during fruit ripening that give fruits their red, purple, pink, or blue color [88]. The fresh date fruits had anthocyanins, while the amount of carotenoids in ripe dates rapidly reduced [89]. Fruits attain good color and flavor when they are adequately supplied with carbohydrates. Moreover, sucrose controls the production of the secondary metabolites carotenoid and anthocyanin, which are responsible for the color of some fruits [90,91]. Exogenous sucrose application enhanced anthocyanin accumulation, accelerating the development of the fruit color of grape berries [92]. Similary, the role of potassium in improving fruit flavor, sugar content, skin color, and reducing fruit skin cracking has been reported in many studies [93,94,95]. A positive linear relationship between color and crop load was found in apples. Fruit thinning decreases the green color and increases the yellow color of yellow apple cultivars and the intensity of the surface color in red cultivars [40]. The findings of the present study showed a higher total sugar content when irrigation water was applied at the 100% ETc level combined with the 5 kg palm^−1^ SOP fertilizer dose, which can be related to a darker/browner fruit color, as the fruits have the lowest L and highest C values. The higher L and ∆E values expressed the lighter/whiter fruit colors, which was observed in fruits which received the lowest dose of SOP fertilizer (2.5 kg palm^−1^). Moreover, bunch thinning also had a significant effect on fruit color. Studying the molecular aspects of the date palm fruit color, [85] reported that the red fruits of cv. Khenezi are produced by the expression of allels VIR^+^/VIR^+^ and VIR^+^/vir^saf^, the yellow fruits of cv. Khalas by vir^saf^/vir^saf^, VIR^IM^/VIR^IM^, VIR^IM^/vir^saf^, and some VIR^+^/VIR^IM^ genotypes and intermediate colors by VIR^+^/VIR^IM^ genotypes, with the action of the VIR^+^/VIR^IM^ genotypes due to the dominant negative inhibition of anthocyanin production.

### 2.6. Fruit Grading and Total Yield

The harvested date palm fruits were categorized into third, second, first, and excellent grades. The third grade fruits are usually supplied to a low class local market, which fetch a minimum return. According to Table 6, there was a significant (*p <* 0.05) difference between all fruit grades, the skin separation percentage, biser fruit percentage, and fruit yield per palm. The maximum third grade fruit percentage was observed at the 80% ETc level and 2.5 kg palm^−1^ SOP fertilizer dose, at all bunch thinning levels. Apart from this, all other treatment combinations statistically behaved alike. The maximum second grade fruit percentage was counted at 80% ETc level + 7.5 kg palm^−1^ SOP fertilizer dose + 10 bunches palm^−1^ thinning level, and was the minimum at the 100% ETc level + 2.5 and 5 kg palm^−1^ SOP fertilizer doses, followed by the 120% ETc level + 2.5 kg palm^−1^ SOP fertilizer dose. Similarly, the maximum first grade fruits were obtained at the 120% ETc level + 5 and 7.5 kg palm^−1^ SOP fertilizer doses, followed by the 100% ETc level combined with all three SOP fertilizer doses. First grade fruits were minimum at 80% and 140% ETc levels at all SOP fertilizer doses and bunch thinning treatment combinations. A more or less similar trend was observed regarding the excellent grade fruit data. Date palm trees irrigated at the 100% ETc level + 5 kg palm^−1^ SOP fertilizer dose + 10 bunches thinning treatment produced the highest percentage of excellent grade fruits, whereas it was lowest at the 80% and 140% ETc levels in combination with all SOP fertilizer and bunch thinning treatments. The skin separation percentage was minimal at the 100% and 120% ETc levels + 5 and 7.5 kg palm^−1^ SOP fertilizer doses + 8–10 bunches palm^−1^ thinning levels, and was highest at the 80% and 140% ETc levels in combination with all SOP fertilizer and bunch thinning treatments. Biser fruits are the date palm fruits that cannot ripen on the tree. Table 6 also indicates that the minimum biser fruit percentage was counted when date palm trees were irrigated at the 100−120% ETc levels at the 5 kg palm^−1^ SOP fertilizer dose and 8−10 bunches palm^−1^ thinning treatments. The biser fruit percentage was higher when plants were irrigated either at 80% or 140% ETc levels, at all SOP fertilizer doses and bunch thinning treatments. The total yield per palm was significantly higher when date palm trees were irrigated at the 100% ETc level, 5 kg palm^−1^ SOP fertilizer dose, and 8–10 bunches palm^−1^ thinning treatments, followed by the 120% ETc level at the same SOP fertilizer dose and bunch thinning treatments. The minimum fruit yield was recorded in all irrigation levels and at the lowest SOP fertilizer dose, i.e., 2.5 kg palm^−1^.

Fruit grading is one of the main steps of date fruit handling after harvest. This procedure can be carried out manually or mechanically [96]. Date fruits are often manually sorted and graded by professionals or guided labor through visual inspection [97]. Date fruit grading is based on the fruit size, ripening, texture, color, and shape. Before the grading process, the damaged fruits, unripe biser fruits, and unfertilized parthenocarpic fruit are usually removed, which have no market value [98]. In the present study, date fruits were graded according to the guidelines provided by [99]. The effect of irrigation water, SOP fertilizer, and thinning treatments of all factors had a significant influence on fruit grading. The lowest and highest levels of irrigation water, lowest dose of SOP fertilizer, and highest bunch thinning palms produced the highest percentage of third grade fruits and lowest percentage of first and excellent grade fruits. Skin puffiness or skin separation also negatively affected the fruit quality of the date palm. It is associated with high temperature and humidity [6] and develops during the ripening of soft date cultivars [100]. In cv. Madjool, that problem was minimized by encouraging early fruit ripening by avoiding excessive irrigation and nitrogen fertilizer [54]. In blueberries, there was a positive correlation between calcium concentrations and fruit firmness, where early calcium foliar application consistently increased the firmness of the fruit [79]. In the present study, the maximum percentage of fruit puffiness was observed at the lowest and highest irrigation levels and was reduced at the optimum irrigation and SOP fertilizer levels. The fruit yield of the date palm is also affected by irrigation water, potassium fertilizer, and crop load [66,101]. Deficit irrigation water applied at 50% ETc resulted in the lowest yield of date palm cv. Succary compared to 100 and 150% ETc treatments [62]. Our findings also showed that the lowest irrigation level and SOP fertilizer dose negatively affected the date palm fruit yield.

### 2.7. Correlation between the Fruit Parameters

**Figure 3:**
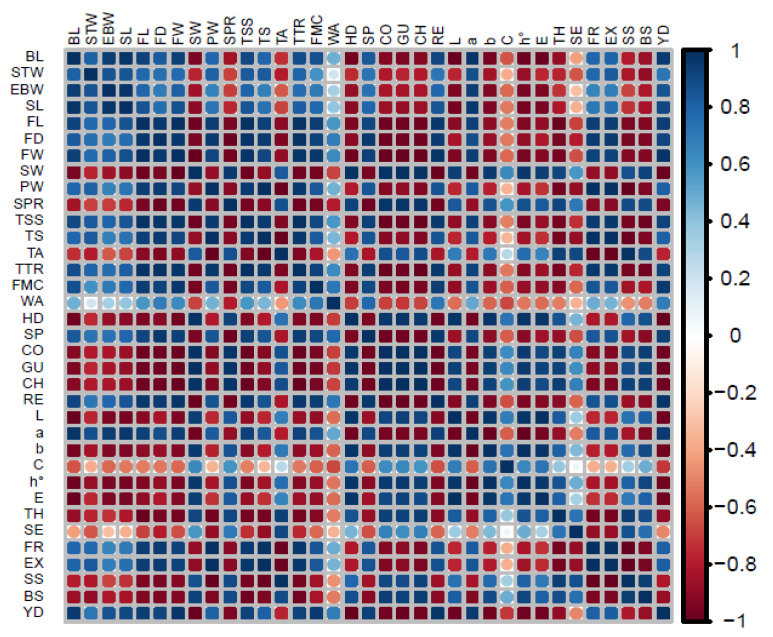
Correlation analysis of bunch and fruit characteristics of date palm cv. Sukary subjected to four irrigation levels, three SOP fertilizer doses, and three bunch thinning levels. Abbreviations: BL: bunch length, STW: stalk width, EMW: empty bunch weight, SL: strand length, FL: fruit length, FD: fruit diameter, FW: fruit weight, SW: seed weight, PW: pulp weight, SPR: seed pulp ratio, TSS: total soluble solids, TS: total sugars, TA: titratable acidity, TTR: total soluble solids and titratable acidity ratio, FMC: fruit moisture content, WA: water activity, HD: hardness, SP: springiness, CO: cohesiveness, GU: gumminess, CH: chewiness, RE: resilience, L: lightness, *a*: greenness-redness, *b*: blueness-yellowness, C: chroma, h°: hue angle, E: color difference, TH: third grade fruits, SE: second grade fruits, FR: first grade fruits, EX: excellent grade fruits, SS: skin separated fruits, BS: biser fruits, and YD: total fruit yield.

In Figure 3, it shown that there was a significant correlation among different bunch and fruit quality and yield parameters of the date palm cv. Sukary. There was a significant positive correlation among BL, STW, EBW, SL, FL, FD, FW, PW, TSS, TS, TTR, FMC, WA, SP, RE, *a*, FR, EX, and YD. These attributes had significant negative correlation with SW, SPR, TA, HD, CO, GU, CH, L, *b*, C, h°, E, TH, SE, SS, and BS. The correlation among fruit texture characteristics indicated that SP and RE had a positive correlation between each other, and had a negative correlation with HD, CO, GU, and CH. The correlation between fruit color parameters showed that there was a significant positive correlation between L and *b*, C, h°, and E, whereas it was negative with *a*. The fruit YD parameter was significantly positively/negatively correlated with different fruit grades, skin puffiness, and unripe fruits. The YD had a significant positive correlation with FR and EX fruit grades, whereas it had a negative correlation with TH, SE, SS, and BS. Similarly, fruit quality parameters such as TSS, TS, TTR, FMC, and WA had a significant positive correlation between each other and had a negative correlation with TA.

## 3. Materials and Methods

### 3.1. Description of Experimental Site

**Figure 4:**
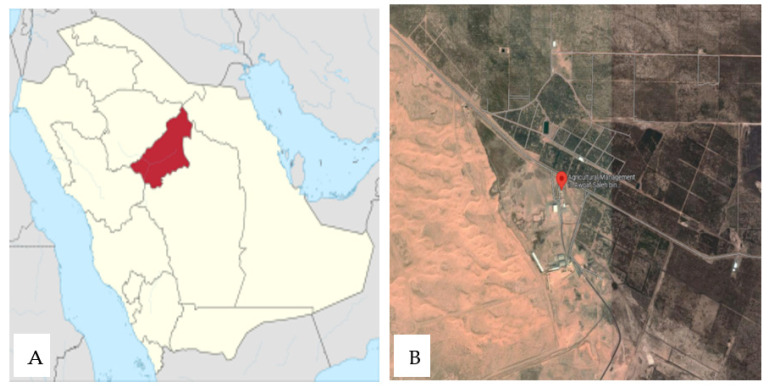
(**A**) The location map of the Al-Qassim region and (**B**) The location map of the experimental site.

The present research was conducted at the private date palm orchard of Endowment Abdul Aziz Saleh Al-Rajhi, Buraydah, in the Central-Eastern part of the Al-Qassim region, Al-Batin Project, Kingdom of Saudi Arabia (latitude 26°19′04.7″ N, longitude 44°07′20.3″ E) (Figure 4A,B). The total area of the date palm orchard comprised of 5466 hectares. The experimental region is a semi-arid area of the kingdom, mostly comprised of sandy hills. The basins between these hills are fertile agricultural lands. This is due to the ease of groundwater extraction from underground limestone and gypsum layers.

### 3.2. Soil and Water Analysis

The soil composite sample was collected at 60 cm depth to analyze the physicochemical properties of the experimental site. The soil samples were air-dried and then passed through a 2 mm sieve for further analysis in the Date Palm Biochemistry Laboratory, King Faisal University, KSA. The soil texture was identified as sandy loam (75.8% sand, 12.5% silt, and 11.7 clay), having 0.58% organic matter, 3.29 dS m^−1^ electrical conductivity (EC), 7.8 pH, 0.07% N, 1.47 ppm P, 0.5 meq L^−1^ K, 32.2 meq L^−1^ Na, 33.3 meq L^−1^ Cl, 14.1 meq L^−1^ Mg, 4.35 meq L^−1^ Ca, 0.22 meq L^−1^ Zn, 0.10 meq L^−1^ Mn, and 1.88 meq L^−1^ Fe. The irrigation water analysis indicated that the pH value was 7.3, EC 2.33 dS m^−1^, total dissolved solids (TDS) 16,473 mg L^−1^, SAR 3.82, RSC −8.72 mg L^−1^, K 0.02 meq L^−1^, Ca 10.5 meq L^−1^, Mg 6.35 meq L^−1^, Na 13.11 meq L^−1^, Cl 20.57 meq L^−1^, Mn 0.47 meq L^−1^, and Fe 2.82 meq L^−1^.

### 3.3. Meteorological Conditions

The meteorological data were acquired using NREL System Advisor Model (SAM) software, version 2020.11.29 (National Renewable Energy Laboratory, Golden, CO, USA). The climate of the Al-Qassim region is characterized by very hot dry summers and mild to cool winters. During the seasons 2020/2021–2021/2022, the coldest month was January with an average temperature of 16–17 °C and average maximum temperatures of 19 °C. The average monthly temperature in February and December was between 18–19 °C. The summer season has the hottest months (June, July, August, and September) and the highest average monthly temperature was recorded in July (40 °C). The average monthly relative humidity during winter ranged between 42–52%, whereas during summer, it ranged between 11–13%. The mean monthly precipitation received during winter was 5.79 mm and no rain was received during summer. The wind speed ranged from 2.30–3.54 km. The average monthly highest solar radiation was recorded in June (341.04–356.29 W m^−2^) and the lowest in December (170.85–171.91 W m^−2^). Similarly, the monthly average air pressure ranged between 14.47–14.78 psi (Table 7).

### 3.4. Experimental Design and Factors Studied

**Figure 5:**
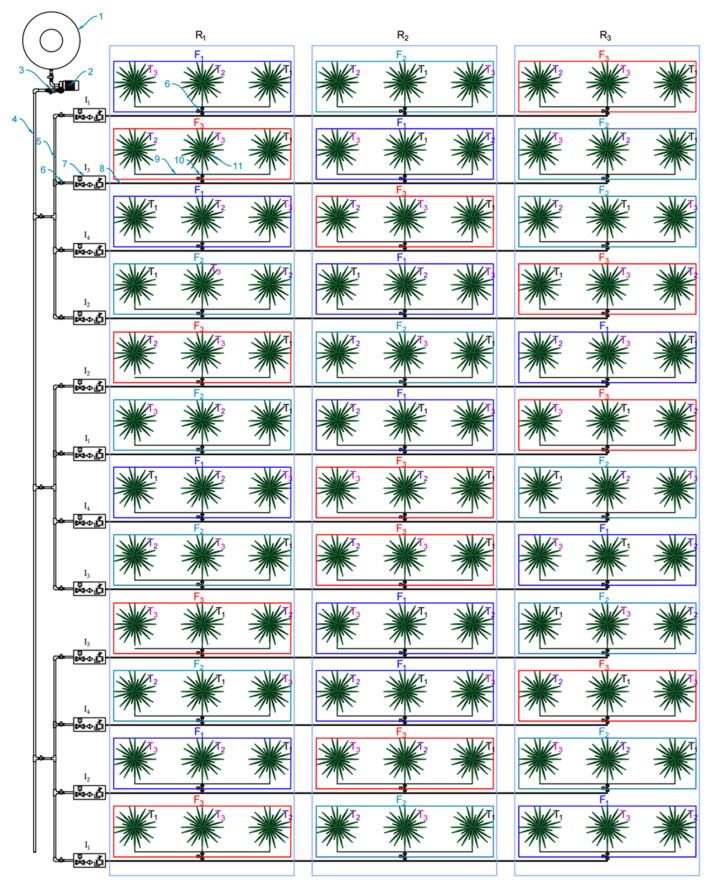
The layout of the experiment. (1) Water tank, (2) irrigation water pump, (3) pressure regulator, (4) main line, (5) sub main line, (6) manual valve, (7) control zone kit, (8) lateral line, (9) distribution line, (10) flow meter, and (11) date palm tree. The layout of the experiment was a three factorial completely randomized block design, i.e., four irrigation levels (I_1_, I_2_, I_3_, and I_4_), three SOP fertilizer doses (F_1_, F_2_, and F_3_), and three bunch thinning levels (T_1_, T_2_, and T_3_), each treatments combination having three replications (R_1_, R_2_, and R_3_).

A two-season study (2020/2021 and 2021/2022) was conducted on 25-year-old, uniform, and healthy date palm trees of cv. Sukary. The selected date palm trees were planted at a distance of 8 × 8 m. Farmyard manure (20 kg palm^−1^) was added in the month of November each year. The experiment was arranged in a completely randomized block design (CRBD) with a three factorial structure having three replications in each treatment combination (Figure 5). The first factor was four irrigation levels (80, 100, 120, and 140% ETc), the second factor was three sulphate of potash (SOP) fertilizer doses (2.5, 5, and 7.5 kg palm^−1^), and the third factor was three bunch thinning treatments (8, 10, and 12 bunches palm^−1^). The bunch thinning was done manually (by hand). The total number of date palm trees included in the present study were 108 (4 × 3 × 3 × 3 = 108). The crop evapotranspiration (ETc) was calculated based on the reference evapotranspiration (ETo) using the following equation:

ETc = Kc × ETo

where Kc is the crop factor, and the average Kc is 0.95.

The ETo was calculated below based on the Penman–Monteith equation [102],

$$\mathrm{ETo}=\frac{0.408\;∆\;\left(R-G\right)+\gamma [900\;u/\left(T+273\right)]\left(e_{s}-e_{a}\right)\;}{∆+\gamma \;\left(1+0.34\;u\right)}$$

where ∆ is the slope vapor pressure curve (kPa °C^−1^), R is the net solar radiation at the surface of the crop (MJ m^−2^ day^−1^), G is the soil heat flux density (MJ m^−2^ day^−1^), γ is the constant psychrometric (kPa °C^−1^), u is the wind speed at 2 m height (m s^−1^), T is the temperature of atmospheric air (°C), e_s_ is the saturation vapor pressure (kPa), and e_a_ is the actual pressure of vapor (kPa), and ETo is the reference evapotranspiration (mm day^−1^) in the study area.

The amount of water required was determined for each date palm tree as below:

$$\mathrm{WR}=\mathrm{ETc}\;\times {\;A}_{\mathrm{ti}}$$

where WR is the amount of required water (m^3^ day^−1^) and $A_{\mathrm{ti}}$ is the irrigation area for a date palm tree (m^2^). The irrigation area was determined based on the light intercepted by the tree canopy as 80% of the shaded area of the date palm tree.

The sulphate of potash (SOP) or potassium sulphate (K_2_SO_4_) is an inorganic white water soluble fertilizer (00:00:50 NPK) containing 50% K_2_O and 17% Sulphur. Due to the absence of chloride, SOP has a much lower salt index; therefore, it is recommended for sandy soils. Each date palm tree received 1.25, 2.5, and 3.75 kg of K_2_O by applying 2.5, 5, and 7.5 kg of SOP, respectively. The traditional agronomic treatments such as irrigation (at 140% ETc), fertilizer (2.5 kg palm^−1^), and bunch thinning (12 bunches palm^−1^) were selected as control, which are already mentioned above. Nitrogen was applied as ammonium nitrate (3 kg palm^−1^), phosphorus was applied as diammonium phosphate (1.2 kg palm^−1^), and calcium was applied as calcium nitrate (150 g palm^−1^). Diammonium phosphate was applied in three split doses (January, February, and April). Ammonium nitrate was divided into four split doses (January, March, April, and May). SOP was applied in six split doses (January, February, March, April, May, and June) and calcium nitrate was applied in three split doses (May, June, and July). The irrigation water was applied using a bubbler irrigation system, and there were four water dispensable bubblers per basin around the date palm trunk. The radius of each basin was 1.5 m from the trunk and the water was applied in the basin using a controlled valve system. The irrigation water was applied on alternating days during the summer (April–October), whereas it was applied on every third day during winter (November–March). The schematic layout of the research study is presented in Figure 5.

### 3.5. On-Site Bubbler Irrigation System

**Figure 6:**
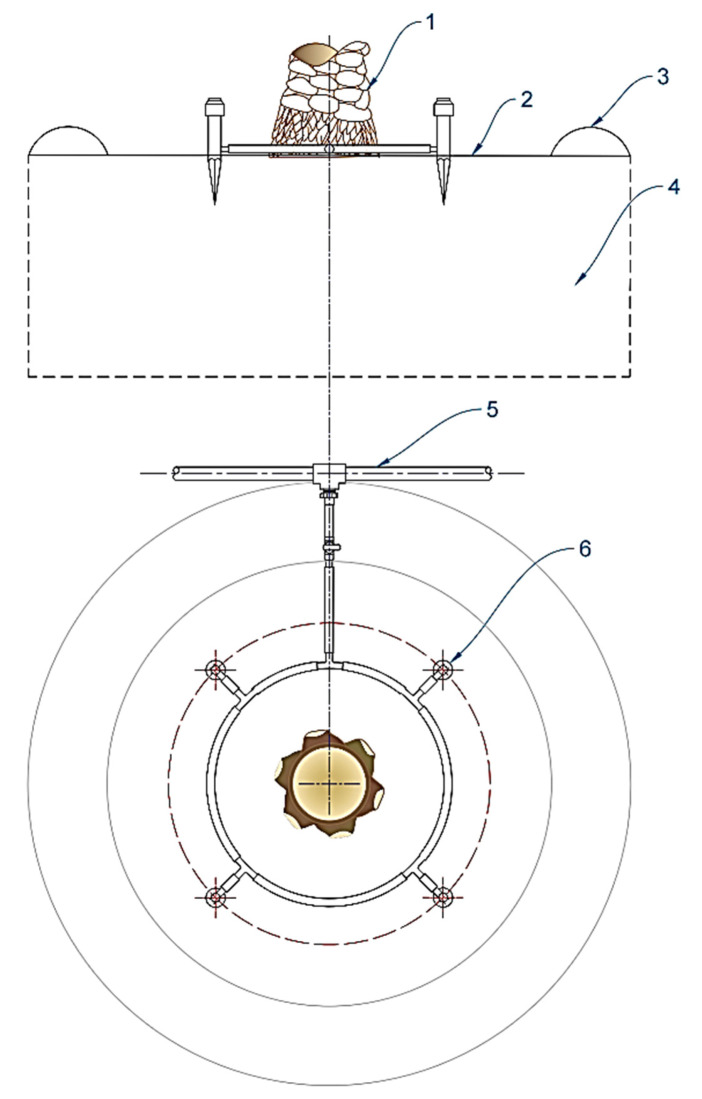
The main components of the bubbler distribution around date palm trees. (1) Date palm tree, (2) soil surface, (3) contour line, al ring, (4) irrigation target zone, (5) distribution line, and (6) pressure-compensating bubbler.

It is a modified version of a drip irrigation system, which consists of a water source, control unit, pumping unit, mixing chamber, mainline and several sub-main lines, laterals, and bubblers. The PVC pipes of the mainline and the sub-main lines were buried, and the hydrants were laid near the surface. The bubblers were above the ground and connected to the laterals with a small flexible tube. Four adjustable bubbler units were used to deliver irrigation water around the trunk of each palm (0–0.12 m^3^ h^−1^), as shown in Figure 6. The bubbler flow rate was adjusted to 0.06 m^3^ h^−1^ by twisting the bubbler head at a pressure of 200 kPa. In order to prevent runoff when irrigation water levels exceeded soil infiltration, a contour line was made around each palm tree. The bubbler head was mounted on a plastic wedge and placed into the ground in a palm basin. A flexible plastic tube with a 1 m length and 7 mm diameter was used to connect the bubbler to the distribution line. The water supply was controlled by solenoid valves in accordance with the irrigation schedules, using four irrigation controllers (Hunter XC-6, Hunter Industries, Inc., San Marcos, CA, USA). Based on the irrigation time, each controller was set for a different amount of ETc-based irrigation water. The irrigation water pressure in the network and the flow rate were controlled using the control zone kit produced by Rain Bird (XCZLF100PRF, Rain Bird Corporation, Tucson, AZ, USA). The control zone kit included a low-flow solenoid valve (0.8 to 37.85 L m^−1^) combined with a large capacity disc filter and high flow pressure regulator (the inlet pressure from 140 to 1030 kPa; the regulated pressure was 210 kPa). It was made specifically to provide filtration, on/off control, and pressure regulation for micro-irrigation systems. The quantitative flow rate of the irrigation water was managed by an automatic controller (Model: LCD-M, SEA, Zhongjiang, China) with a water flow sensor (Model: RPi 20-YF-S201, Guangzhou, China) [12,13].

### 3.6. Fruit Bunch Parameters

**Figure 7:**
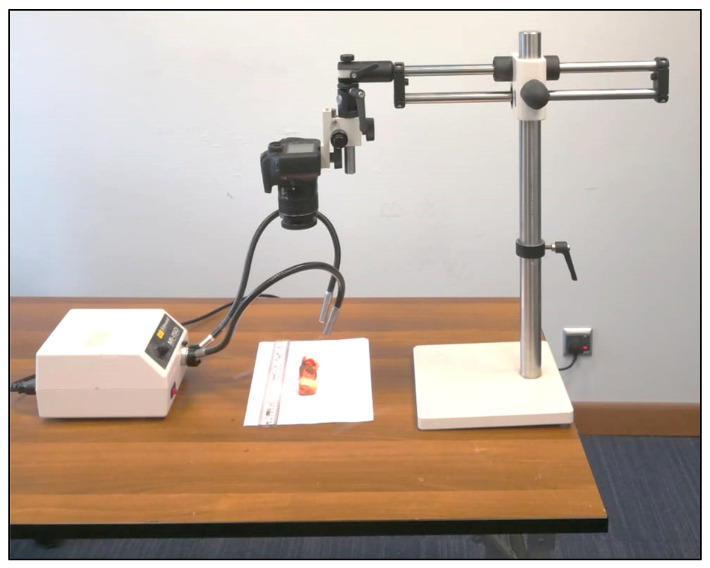
Image of the machine-vision system used for determining skin separation percentage of date fruits.

**Figure 8:**
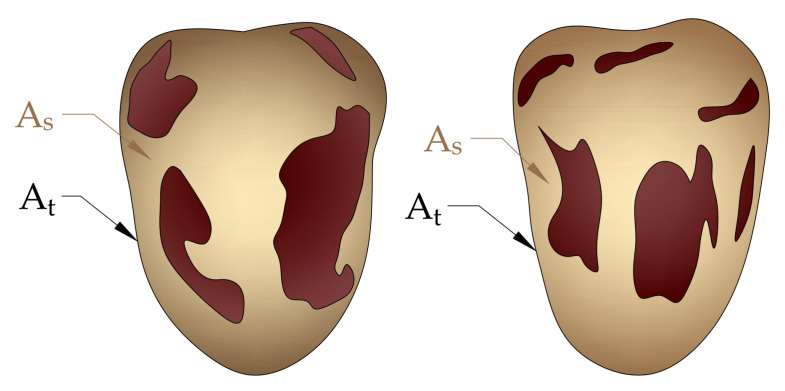
Projected area of the tested date fruit; (A_s_) the separated skin projected area and (A_t_) the total projected area of the tested date fruit.

After harvest, each fruit bunch was carefully tagged and brought to the Date Palm Biochemistry laboratory, King Faisal University for physicochemical analysis. Fruit bunch length (cm) and strand length (cm) were measured with the help of measuring tape, whereas bunch stalk width (cm), fruit length and width were measured using a digital Vernier caliper. Twenty fruits per bunch were randomly selected to determine fruit, seed, and pulp weight using an electronic balance (Sartorius Lab Instruments GmbH and Co., Göttingen, Lower Saxony, Germany). Empty bunch weight (g) and total fruit yield per palm were measured using a digital electronic balance, scale 10 kg (US Solid Electronic Precision Balance, Cleveland, OH, USA). The percentage of biser fruits per bunch was calculated by counting the total number of unripe fruits (biser) per bunch divided by the total number of fruits per bunch, multiplied by a hundred. Date palm fruit were graded into first grade, second grade, third grade, and excellent grade fruits according to the guideline and standards developed by the Ministry of Environment, Water and Agriculture, KSA for local and international trade [99]. After harvest, tamr fruits having puffy skin (exocarp) and detached from the mesocarp were labeled as skin separated fruits and their percentage was calculated. The skin separation percentage of date fruits were determined using the image processing method described by [103,104,105]. A LED light source with a power of 20 W at a fixed height of 10 cm and a digital camera (Model: Canon 4000D) were used to capture the images of the tested date fruits. The captured image of the date fruit were processed using the open-source image processing software (ImageJ/Fiji 1.46, LOCI, University of Wisconsin, Madison, USA) to determine the separated skin area (Figure 7). Figure 8 shows the projected area of the tested date fruit.

The skin separation percentage of the date fruits was determined by the following formula:

$$Skin\;separation\;\left(\%\right)=\frac{A_{s}}{A_{t}}\times 100$$

where *A_s_* is the separated skin projected area and *A_t_* is the total projected area of the tested date fruit.

### 3.7. Fruit Quality Parameters

The total soluble solids (TSS) of the fruits were determined using a handheld digital refractometer (Model: DR 303, Richmond Scientific Ltd. Chorley, Lancashire, UK). The titratable acidity of the date palm was determined by titrating 10 mL of juice against 0.1 N NaOH of alkali solution, using phenolphthalein as an indicator until the pink color persisted for at least 15 s, and was expressed in percentage malic acid [106]. The TSS and titratable acidity ratio was also calculated. The total sugar content was measured using the anthrone method. Fresh date (0.5 g) was boiled with 10 mL of ethanol (80%) and filtered. The volume of the filtrate was made up to 50 mL. The reaction mixture was prepared by mixing 1 mL filtrate and 5 mL anthrone reagent, and placed into a water bath for 15 min at 100 °C. The reaction mixture was then taken from the water bath and left at room temperature for 20 min, and then subjected to an absorbance measurement at 630 nm in a spectrophotometer (Genesys 20, Thermo Scientific, Waltham, MA, USA). The total sugar content was calculated using a standard blank sample relative curve and the absorbance of the date sample [107]. The water activity of fruits was measured using a water activity device (Model: Aqualab Series 3, Decagon Devices, Inc., Pullman, USA). The fruit moisture content was determined by drying a sample of 25 g in a Petri dish under vacuum oven (BINDER™ Series E Classic, Fisher Scientific, Waltham, MA, USA) to a constant weight at 70 °C for 72 h to drive off the water from the sample. The moisture content was determined by the following formula:

$$\mathrm{Moisture}\;\mathrm{content}\;\left(\%\right)=\frac{W2-W3}{W2-W1}\times 100$$

where W1 is the weight of the Petri dish, W2 is the weight of the Petri dish and sample before drying, and W3 is the weight of the Petri dish and sample after drying.

A Hunter lab Color Quest -45/0 LAV color difference meter (Hunter Associates Laboratory Inc., USA) was used to measure the color of the fruit based on the *L*, *a*, and *b* color system [108]. The *L* value is the lightness factor that gives values ranging from zero for black to 100 for white. Chroma (*C*), hue angle (*h*°), and the color difference (∆*E*) were calculated according to the following equations:

$$C=\sqrt{a^{2}+b^{2}}$$

$$h^{\circ }=arc\;tan\frac{b}{a}$$

$$\Delta E=\sqrt{{\left(L_{2}-L_{1}\right)}^{2}+{\left(a_{2}-a_{1}\right)}^{2}+{\left(b_{2}-b_{1}\right)}^{2}}$$

where *C* is Chroma, *h°* is hue angle (degree), *a* is greenness-redness, *b* is blueness-yellowness, *L* is fruit lightness, and ∆*E* is color difference of the fruit.

The fruit texture profile (hardness, springiness, cohesiveness, gumminess, chewiness, and resilience) was recorded at room temperature using a digital texture analyzer (TA. XTPlus Connect, Texture Technologies Corp. and Stable Micro Systems, Ltd., Hamilton, MA, USA) equipped with a force capacity of 50 kg f (500 N), force resolution of 0.1 g, load cells of 0.5, 5, 30, 50 kg f, speed range from 0.01–40 mm s^−1^, maximum aperture of 370 mm/590 mm, distance resolution of 0.001 mm, and data acquisition rate of 2000 pps.

### 3.8. Statistical Analysis

The experiment was designed on a three factorial completely randomized block design (CRBD), having three replicated trees in each treatment. The first factor was irrigation levels: (1) 80% ETc, (2) 100% ETc, (3) 120% ETc, and (4) 140% Etc; the second factor was SOP fertilizer doses: (1) 2.5 kg palm^−1^, (2) 5 kg palm^−1^, and (3) 7.5 kg palm^−1^; and the third factor was bunch thinning: (1) 8 bunches palm^−1^, (2) 10 bunches palm^−1^, and (3) 12 bunches palm^−1^. One hundred and eight date palm trees were included in the study. The analysis of variance (ANOVA) of the collected data was performed using the SPSS statistical program (SPSS Inc., Chicago, IL, USA) and a difference between the means at 5% probability (*p <* 0.05) was considered significant. The statistical differences between treatment means of the data were compared by a Least Significant Difference (LSD) test at 5% level of probability (*p <* 0.05).

## 4. Conclusions

The present study demonstrated differences in the fruit yield, quality, texture, color, and skin separation attributes of the date palm cv. Sukary in response to irrigation levels, potassium fertilizer application doses, fruit bunch thinning levels, and their interactions. The research findings showed a promising performance of cv. Sukary under agronomic conditions of 100% ETc level of irrigation, medium (5 kg palm^−1^) SOP fertilizer applied to sandy loam soils, and retaining 8–10 fruit bunches palm^−1^, for improved crop productivity and associated traits, fruit quality, texture, color, and skin separation characteristics. For better inferences, however, it is suggested that these findings be validated under different soils and environmental conditions. Future research may also incorporate other factors, such as the interaction between air temperature and relative humidity with skin texture and mechanical properties, and the combined effect of potassium and calcium on the skin separation properties of the fruit. Micro details about the upregulation/downregulation of different enzymes involved during skin separation can also be studied.

## Figures and Tables

**Table 1:** Effects of irrigation levels, SOP fertilizer doses, and bunch thinning levels on the bunch length, stalk width, empty bunch weight, and strand length of date palm cv. Sukary. Each mean data represents the averages from two seasons 2020/2021–2021/2022.

Irrigation Levels	SOP Fertilizer Doses	Thinning Levels (Bunches Palm^−1^)	Bunch Length (cm)	Stalk Width (cm)	Empty Bunch Weight (g)	Strand Length (cm)
I_1_ (80% ETc)	F_1_ (2.5 kg palm^−1^)	T_1_ (8 Bunches)	67.66 ^BG^	4.43 ^AD^	1.66 ^AC^	11.46 ^BG^
		T_2_ (10 Bunches)	64.00 ^G^	4.26 ^BE^	1.16 ^D^	11.46 ^BG^
		T_3_ (12 Bunches)	64.00 ^G^	3.3 ^F^	1.33 ^BD^	11.20 ^EG^
	F_2_ (5 kg palm^−1^)	T_1_ (8 Bunches)	73.33 ^AG^	4.70 ^AC^	1.60 ^AD^	12.23 ^BE^
		T_2_ (10 Bunches)	73.33 ^AG^	4.70 ^AC^	1.60 ^AD^	11.46 ^BG^
		T_3_ (12 Bunches)	73.00 ^AG^	4.56 ^AD^	1.20 ^CD^	11.33 ^CG^
	F_3_ (7.5 kg palm^−1^)	T_1_ (8 Bunches)	77.33 ^AE^	4.56 ^AD^	1.60 ^AD^	12.30 ^BD^
		T_2_ (10 Bunches)	77.33 ^AE^	4.20 ^BE^	1.60 ^AD^	12.13 ^BE^
		T_3_ (12 Bunches)	77.00 ^AF^	4.03 ^CF^	1.40 ^BD^	11.96 ^BF^
I_2_ (100% ETc)	F_1_ (2.5 kg palm^−1^)	T_1_ (8 Bunches)	70.66 ^AG^	4.83 ^AB^	1.56 ^AD^	11.77 ^BG^
		T_2_ (10 Bunches)	66.33 ^DG^	4.66 ^AC^	1.53 ^AD^	11.46 ^BG^
		T_3_ (12 Bunches)	65.33 ^EG^	4.60 ^AD^	1.20 ^CD^	11.30 ^DG^
	F_2_ (5 kg palm^−1^)	T_1_ (8 Bunches)	82.66 ^A^	5.16 ^A^	1.90 ^A^	13.43 ^A^
		T_2_ (10 Bunches)	80.00 ^AB^	5.06 ^A^	1.70 ^AB^	12.43 ^AC^
		T_3_ (12 Bunches)	80.00 ^AB^	4.83 ^AB^	1.70 ^AB^	12.30 ^BE^
	F_3_ (7.5 kg palm^−1^)	T_1_ (8 Bunches)	79.00 ^AD^	4.60 ^AD^	1.53 ^AD^	12.40 ^AD^
		T_2_ (10 Bunches)	79.00 ^AD^	4.50 ^AD^	1.50 ^AD^	12.10 ^BE^
		T_3_ (12 Bunches)	76.66 ^AG^	4.26 ^BE^	1.33 ^BD^	12.07 ^BE^
I_3_ (120% ETc)	F_1_ (2.5 kg palm^−1^)	T_1_ (8 Bunches)	72.00 ^AG^	4.46 ^AD^	1.33 ^BD^	11.33 ^CG^
		T_2_ (10 Bunches)	70.66 ^AG^	4.30 ^BE^	1.30 ^BD^	10.90 ^FG^
		T_3_ (12 Bunches)	68.33 ^BG^	4.26 ^BE^	1.20 ^CD^	10.80 ^G^
	F_2_ (5 kg palm^−1^)	T_1_ (8 Bunches)	77.00 ^AE^	4.56 ^AD^	1.63 ^AD^	12.40 ^AD^
		T_2_ (10 Bunches)	80.33 ^AB^	4.50 ^AD^	1.56 ^AD^	11.76 ^BG^
		T_3_ (12 Bunches)	75.66 ^AG^	4.00 ^CF^	1.53 ^AD^	11.46 ^BG^
	F_3_ (7.5 kg palm^−1^)	T_1_ (8 Bunches)	79.33 ^AC^	4.23 ^BE^	1.53 ^AD^	11.90 ^BG^
		T_2_ (10 Bunches)	77.33 ^AE^	4.10 ^BF^	1.46 ^AD^	11.90 ^BG^
		T_3_ (12 Bunches)	74.66 ^AG^	3.90 ^DF^	1.36 ^BD^	11.26 ^EG^
I_4_ (140% ETc)	F_1_ (2.5 kg palm^−1^)	T_1_ (8 Bunches)	70.33 ^AG^	4.30 ^BE^	1.40 ^BD^	11.40 ^BG^
		T_2_ (10 Bunches)	66.66 ^CG^	4.10 ^BF^	1.20 ^CD^	11.40 ^G^
		T_3_ (12 Bunches)	64.33 ^FG^	4.10 ^BF^	1.16 ^D^	11.33 ^CG^
	F_2_ (5 kg palm^−1^)	T_1_ (8 Bunches)	73.33 ^AG^	4.46 ^AD^	1.70 ^AB^	11.83 ^BG^
		T_2_ (10 Bunches)	73.33 ^AG^	4.46 ^AD^	1.56 ^AD^	11.76 ^BG^
		T_3_ (12 Bunches)	73.33 ^AG^	4.26 ^BF^	1.56 ^AD^	11.73 ^BG^
	F_3_ (7.5 kg palm^−1^)	T_1_ (8 Bunches)	77.33 ^AE^	4.23 ^BE^	1.36 ^BD^	11.73 ^BG^
		T_2_ (10 Bunches)	75.66 ^AG^	3.90 ^DF^	1.33 ^BD^	11.60 ^BG^
		T_3_ (12 Bunches)	64.33 ^FG^	3.43 ^F^	1.30 ^BD^	11.60 ^BG^

Different letters in a row show significant differences referring to treatment means. The ANOVA was constructed using a three factorial completely randomized block design, i.e., four irrigation levels (I_1_, I_2_, I_3_, and I_4_), three SOP fertilizer doses (F_1_, F_2_, and F_3_), and three bunch thinning levels (T_1_, T_2_, and T_3_). The significant data were subjected to a least significant difference (LSD) test for mean comparison at 5% level of probability (*p <* 0.05).

**Table 2:** Effects of irrigation levels, SOP fertilizer doses, and bunch thinning levels on the fruit length, fruit width, fruit weight, seed weight, pulp weight, and seed pulp ratio of date palm cv. Sukary. Each mean data represents the averages from two seasons, 2020/2021–2021/2022.

Irrigation Levels	SOP Fertilizer Doses	Thinning Levels (Bunches Palm^−1^)	Fruit Length (cm)	Fruit Width (cm)	Fruit Weight (g)	Seed Weight (g)	Pulp Weight (g)	Seed: Pulp Ratio
I_1_ (80% ETc)	F_1_ (2.5 kg palm^−1^)	T_1_ (8 Bunches)	2.21 ^PQ^	1.88 ^HI^	8.69 ^F^	2.23 ^A^	6.97 ^FG^	0.30 ^A^
		T_2_ (10 Bunches)	2.16 ^PQ^	1.86 ^HI^	7.84 ^F^	2.20 ^A^	7.05 ^FG^	0.31 ^A^
		T_3_ (12 Bunches)	2.07 ^Q^	1.74 ^HI^	7.60 ^F^	2.23 ^A^	7.10 ^FG^	0.31 ^A^
	F_2_ (5 kg palm^−1^)	T_1_ (8 Bunches)	2.69 ^KM^	1.82 ^HI^	8.94 ^EF^	2.19 ^A^	6.82 ^FG^	0.30 ^A^
		T_2_ (10 Bunches)	2.75 ^IM^	1.95 ^FH^	9.10 ^EF^	2.14 ^AC^	6.96 ^FG^	0.31 ^A^
		T_3_ (12 Bunches)	2.62 ^IN^	1.90 ^GI^	8.87 ^EF^	2.07 ^AF^	6.86 ^FG^	0.30 ^A^
	F_3_ (7.5 kg palm^−1^)	T_1_ (8 Bunches)	2.72 ^JM^	1.70 ^I^	8.69 ^F^	2.28 ^A^	7.18 ^FG^	0.31 ^A^
		T_2_ (10 Bunches)	2.83 ^FL^	1.82 ^HI^	9.16 ^EF^	2.16 ^AB^	6.99 ^FG^	0.30 ^A^
		T_3_ (12 Bunches)	2.69 ^KM^	1.70 ^I^	8.54 ^F^	2.14 ^AC^	8.60 ^DF^	0.26 ^A^
I_2_ (100% ETc)	F_1_ (2.5 kg palm^−1^)	T_1_ (8 Bunches)	2.83 ^GL^	2.30 ^AE^	10.74 ^DE^	2.05 ^AF^	11.88 ^A^	0.18 ^B^
		T_2_ (10 Bunches)	2.85 ^GL^	2.26 ^AE^	12.26 ^BD^	2.05 ^AF^	11.57 ^AB^	0.18 ^BC^
		T_3_ (12 Bunches)	2.53 ^MO^	2.24 ^AE^	9.33 ^EF^	2.25 ^A^	11.49 ^AB^	0.17 ^BC^
	F_2_ (5 kg palm^−1^)	T_1_ (8 Bunches)	3.36 ^A^	2.40 ^A^	14.35 ^A^	1.15 ^K^	12.13 ^A^	0.12 ^C^
		T_2_ (10 Bunches)	3.38 ^A^	2.41 ^AE^	14.59 ^A^	1.14 ^K^	11.87 ^A^	0.14 ^BC^
		T_3_ (12 Bunches)	3.36 ^A^	2.39 ^AB^	14.12 ^AB^	1.15 ^K^	12.44 ^A^	0.14 ^BC^
	F_3_ (7.5 kg palm^−1^)	T_1_ (8 Bunches)	3.34 ^AB^	2.37 ^AC^	14.55 ^A^	1.15 ^K^	12.44 ^A^	0.14 ^BC^
		T_2_ (10 Bunches)	3.37 ^A^	2.36 ^AD^	13.53 ^AB^	1.16 ^K^	11.63 ^AB^	0.14 ^BC^
		T_3_ (12 Bunches)	3.29 ^AC^	2.29 ^AE^	13.49 ^AB^	1.13 ^K^	11.74 ^AB^	0.15 ^BC^
I_3_ (120% ETc)	F_1_ (2.5 kg palm^−1^)	T_1_ (8 Bunches)	2.99 ^DJ^	2.21 ^AE^	10.71 ^DE^	2.25 ^A^	11.94 ^A^	0.18 ^BC^
		T_2_ (10 Bunches)	3.00 ^DI^	2.28 ^AE^	9.47 ^EF^	2.15 ^AC^	11.51 ^AB^	0.19 ^B^
		T_3_ (12 Bunches)	2.96 ^EK^	2.21 ^AE^	9.00 ^EF^	2.14 ^AC^	11.03 ^AB^	0.15 ^BC^
	F_2_ (5 kg palm^−1^)	T_1_ (8 Bunches)	3.25 ^AD^	2.23 ^AE^	13.69 ^AB^	1.50 ^HJ^	11.02 ^AB^	0.16 ^BC^
		T_2_ (10 Bunches)	3.21 ^AE^	2.23 ^AE^	13.17 ^AC^	1.21 ^JK^	10.74 ^AC^	0.15 ^BC^
		T_3_ (12 Bunches)	3.20 ^AE^	2.21 ^AE^	12.83 ^AC^	1.15 ^K^	10.64 ^AC^	0.15 ^BC^
	F_3_ (7.5 kg palm^−1^)	T_1_ (8 Bunches)	3.07 ^BG^	2.21 ^AE^	14.07 ^AB^	1.40 ^IK^	10.98 ^AB^	0.15 ^BC^
		T_2_ (10 Bunches)	3.15 ^AF^	2.23 ^AE^	13.74 ^AB^	1.50 ^HJ^	9.99 ^BD^	0.15 ^BC^
		T_3_ (12 Bunches)	3.03 ^CH^	2.19 ^AE^	13.70 ^AB^	1.61 ^GI^	9.10 ^CE^	0.17 ^BC^
I_4_ (140% ETc)	F_1_ (2.5 kg palm^−1^)	T_1_ (8 Bunches)	2.76 ^HM^	2.15 ^DF^	9.12 ^EF^	2.10 ^AE^	7.78 ^EG^	0.19 ^B^
		T_2_ (10 Bunches)	2.73 ^IM^	2.12 ^EG^	9.25 ^EF^	1.86 ^BG^	6.68 ^G^	0.19 ^B^
		T_3_ (12 Bunches)	2.64 ^LN^	2.10 ^EG^	8.95 ^EF^	1.79 ^EH^	6.46 ^G^	0.17 ^BC^
	F_2_ (5 kg palm^−1^)	T_1_ (8 Bunches)	2.52 ^MO^	2.17 ^BE^	11.49 ^CD^	1.82 ^CH^	7.85 ^EG^	0.14 ^BC^
		T_2_ (10 Bunches)	2.37 ^NP^	2.16 ^CF^	9.39 ^EF^	1.68 ^GI^	7.55 ^EG^	0.15 ^BC^
		T_3_ (12 Bunches)	2.28 ^OQ^	2.15 ^DF^	9.28 ^EF^	1.61 ^GI^	6.28 ^G^	0.15 ^BC^
	F_3_ (7.5 kg palm^−1^)	T_1_ (8 Bunches)	3.14 ^AF^	2.18 ^BE^	12.84 ^AC^	1.74 ^FH^	7.53 ^EG^	0.15 ^BC^
		T_2_ (10 Bunches)	3.01 ^CI^	2.22 ^AE^	12.42 ^BD^	1.66 ^GI^	8.18 ^DG^	0.17 ^BC^
		T_3_ (12 Bunches)	3.01 ^CI^	2.17 ^CF^	12.39 ^BD^	1.80 ^DH^	7.80 ^EG^	0.16 ^BC^

Different letters in a row show significant differences referring to treatment means. The ANOVA was constructed using a three factorial completely randomized block design, i.e., four irrigation levels (I_1_, I_2_, I_3_, and I_4_), three SOP fertilizer doses (F_1_, F_2_, and F_3_), and three bunch thinning levels (T_1_, T_2_, and T_3_). The significant data were subjected to a least significant difference (LSD) test for mean comparison at 5% level of probability (*p <* 0.05).

**Table 3:** Effects of irrigation levels, SOP fertilizer doses, and bunch thinning levels on the total soluble solids (TSS), titratable acidity, total soluble solids and titratable acidity ratio, total sugars, moisture content, and water activity of date palm cv. Sukary. Each mean data represents the averages from two seasons 2020/2021–2021/2022.

Irrigation Levels	SOP Fertilizer Doses	Thinning Levels (Bunches Palm^−1^)	TSS (Brix)	Titratable Acidity (%)	TSS: Titratable Acidity ratio	Total Sugars (%)	Moisture Content (%)	Water Activity (a_w_)
I_1_ (80% ETc)	F_1_ (2.5 kg palm^−1^)	T_1_ (8 Bunches)	59.00 ^JK^	0.17 ^C^	347.05 ^AB^	61.66 ^I^	14.80 ^EF^	0.435 ^A^
		T_2_ (10 Bunches)	58.00 ^K^	0.17 ^C^	341.01 ^C^	61.66 ^I^	14.80 ^EF^	0.434 ^A^
		T_3_ (12 Bunches)	59.00 ^JK^	0.17 ^C^	347.05 ^BC^	63.33 ^FI^	14.80 ^EF^	0.434 ^A^
	F_2_ (5 kg palm^−1^)	T_1_ (8 Bunches)	60.00 ^GK^	0.17 ^C^	353.29 ^BC^	63.33 ^FI^	14.80 ^EF^	0.419 ^A^
		T_2_ (10 Bunches)	58.00 ^K^	0.17 ^C^	345.05 ^C^	63.00 ^GI^	14.91 ^EF^	0.420 ^A^
		T_3_ (12 Bunches)	59.33 ^IK^	0.17 ^C^	349.00 ^BC^	63.00 ^GI^	14.67 ^F^	0.429 ^A^
	F_3_ (7.5 kg palm^−1^)	T_1_ (8 Bunches)	59.66 ^HK^	0.18 ^C^	330.83 ^CD^	63.00 ^GI^	14.60 ^F^	0.432 ^A^
		T_2_ (10 Bunches)	59.66 ^HK^	0.16 ^C^	372.87 ^AB^	61.66 ^I^	14.67 ^F^	0.429 ^A^
		T_3_ (12 Bunches)	60.33 ^FK^	0.17 ^C^	353.52 ^BC^	62.66 ^HI^	14.80 ^EF^	0.432 ^A^
I_2_ (100% ETc)	F_1_ (2.5 kg palm^−1^)	T_1_ (8 Bunches)	63.66 ^CH^	0.37 ^A^	169.05 ^Q^	71.00 ^B^	14.80 ^EF^	0.456 ^A^
		T_2_ (10 Bunches)	67.00 ^C^	0.37 ^A^	181.08 ^OQ^	70.00 ^BD^	15.02 ^DF^	0.435 ^A^
		T_3_ (12 Bunches)	65.33 ^CE^	0.37 ^A^	178.05 ^OQ^	70.00 ^BD^	15.83 ^CF^	0.434 ^A^
	F_2_ (5 kg palm^−1^)	T_1_ (8 Bunches)	76.66 ^A^	0.37 ^A^	207.18 ^KM^	77.33 ^A^	17.59 ^AD^	0.444 ^A^
		T_2_ (10 Bunches)	76.66 ^A^	0.37 ^A^	207.18 ^KM^	77.00 ^A^	16.02 ^BE^	0.437 ^A^
		T_3_ (12 Bunches)	74.66 ^AB^	0.37 ^A^	201.78 ^LN^	77.00 ^A^	17.31 ^AC^	0.436 ^A^
	F_3_ (7.5 kg palm^−1^)	T_1_ (8 Bunches)	73.66 ^AB^	0.37 ^A^	199.08 ^NO^	76.66 ^A^	18.56 ^AB^	0.440 ^A^
		T_2_ (10 Bunches)	73.66 ^AB^	0.37 ^A^	198.18 ^NO^	77.00 ^A^	17.34 ^AE^	0.442 ^A^
		T_3_ (12 Bunches)	73.33 ^AB^	0.37 ^A^	198.18 ^NO^	77.00 ^A^	17.18 ^AF^	0.440 ^A^
I_3_ (120% ETc)	F_1_ (2.5 kg palm^−1^)	T_1_ (8 Bunches)	62.66 ^DJ^	0.25 ^B^	250.64 ^HJ^	68.33 ^BE^	16.75 ^AF^	0.443 ^A^
		T_2_ (10 Bunches)	64.33 ^CE^	0.26 ^B^	247.42 ^JK^	68.33 ^BE^	16.97 ^AF^	0.425 ^A^
		T_3_ (12 Bunches)	64.00 ^CG^	0.25 ^B^	250.04 ^HJ^	68.66 ^BE^	15.27 ^DF^	0.456 ^A^
	F_2_ (5 kg palm^−1^)	T_1_ (8 Bunches)	74.00 ^AB^	0.25 ^B^	296.00 ^EF^	75.00 ^A^	16.52 ^AF^	0.457 ^A^
		T_2_ (10 Bunches)	74.00 ^AB^	0.26 ^B^	284.61 ^FG^	75.33 ^A^	16.78 ^AF^	0.456 ^A^
		T_3_ (12 Bunches)	72.33 ^B^	0.25 ^B^	289.32 ^FG^	75.66 ^A^	16.45 ^AF^	0.447 ^A^
	F_3_ (7.5 kg palm^−1^)	T_1_ (8 Bunches)	67.33 ^C^	0.26 ^B^	258.96 ^HJ^	70.00 ^BD^	18.62 ^AB^	0.442 ^A^
		T_2_ (10 Bunches)	66.66 ^CD^	0.25 ^B^	266.64 ^GH^	71.00 ^B^	18.56 ^AB^	0.449 ^A^
		T_3_ (12 Bunches)	66.00 ^CD^	0.26 ^B^	253.84 ^HJ^	69.66 ^BD^	17.35 ^AE^	0.449 ^A^
I_4_ (140% ETc)	F_1_ (2.5 kg palm^−1^)	T_1_ (8 Bunches)	66.66 ^CD^	0.18 ^C^	370.33 ^AB^	70.66 ^BC^	14.91 ^EF^	0.457 ^A^
		T_2_ (10 Bunches)	65.66 ^CE^	0.18 ^C^	364.77 ^B^	69.00 ^BE^	14.80 ^EF^	0.456 ^A^
		T_3_ (12 Bunches)	63.33 ^CI^	0.19 ^C^	333.31 ^CD^	67.66 ^BE^	14.80 ^EF^	0.457 ^A^
	F_2_ (5 kg palm^−1^)	T_1_ (8 Bunches)	61.66 ^EK^	0.16 ^C^	385.37 ^A^	68.33 ^BE^	16.90 ^AF^	0.457 ^A^
		T_2_ (10 Bunches)	62.66 ^DJ^	0.18 ^C^	348.11 ^BC^	67.00 ^CF^	14.80 ^BF^	0.457 ^A^
		T_3_ (12 Bunches)	60.33 ^FK^	0.19 ^C^	317.52 ^D^	65.66 ^EH^	16.40 ^AF^	0.457 ^A^
	F_3_ (7.5 kg palm^−1^)	T_1_ (8 Bunches)	60.33 ^FK^	0.19 ^C^	317.52 ^D^	65.66 ^EH^	18.53 ^AB^	0.457 ^A^
		T_2_ (10 Bunches)	60.66 ^FK^	0.19 ^C^	319.26 ^D^	67.33 ^BE^	18.73 ^A^	0.456 ^A^
		T_3_ (12 Bunches)	60.66 ^FK^	0.20 ^C^	303.30 ^E^	66.66 ^DG^	18.46 ^AB^	0.456 ^A^

Different letters in a row show significant differences referring to treatment means. The ANOVA was constructed using a three factorial completely randomized block design, i.e., four irrigation levels (I_1_, I_2_, I_3_, and I_4_), three SOP fertilizer doses (F_1_, F_2_, and F_3_), and three bunch thinning levels (T_1_, T_2_, and T_3_). The significant data were subjected to a least significant difference (LSD) test for mean comparison at 5% level of probability (*p <* 0.05).

**Table 4:** Effects of irrigation levels, SOP fertilizer doses, and bunch thinning levels on the hardness, springiness, cohesiveness, gumminess, chewiness, and resilience of date palm cv. Sukary. Each mean data represents the averages from two seasons, 2020/2021–2021/2022.

Irrigation Levels	SOP Fertilizer Doses	Thinning Levels (Bunches Palm^−1^)	Hardness (N)	Springiness	Cohesiveness	Gumminess	Chewiness	Resilience
I_1_ (80% ETc)	F_1_ (2.5 kg palm^−1^)	T_1_ (8 Bunches)	18.48 ^AB^	0.686 ^FI^	0.710 ^A^	1278.6 ^A^	779.29 ^AB^	0.129 ^I^
		T_2_ (10 Bunches)	18.08 ^AC^	0.693 ^FI^	0.669 ^AB^	1035.3 ^AE^	895.72 ^A^	0.129 ^I^
		T_3_ (12 Bunches)	21.12 ^A^	0.638 ^I^	0.669 ^AB^	1067. 7 ^AD^	812.3 ^BF^	0.143 ^HI^
	F_2_ (5 kg palm^−1^)	T_1_ (8 Bunches)	11.46 ^CJ^	0.686 ^FI^	0.638 ^AC^	1102. 3 ^AC^	725.78 ^AC^	0.148 ^GI^
		T_2_ (10 Bunches)	11.00 ^DJ^	0.678 ^GI^	0.648 ^AD^	1125.8 ^AB^	748.56 ^AC^	0.149 ^GI^
		T_3_ (12 Bunches)	17.13 ^AD^	0.638 ^I^	0.645 ^AE^	1084.6 ^AC^	757.47 ^AB^	0.146 ^HI^
	F_3_ (7.5 kg palm^−1^)	T_1_ (8 Bunches)	14.61 ^BF^	0.638 ^I^	0.660 ^AE^	929. 0 ^BF^	663.26 ^AD^	0.145 ^HI^
		T_2_ (10 Bunches)	15.50 ^AE^	0.677 ^HI^	0.657 ^AC^	944.4 ^AF^	695.02 ^AC^	0.143 ^HI^
		T_3_ (12 Bunches)	18.01 ^AC^	0.678 ^GI^	0.645 ^AE^	912. 3 ^BF^	668.13 ^AD^	0.146 ^HI^
I_2_ (100% ETc)	F_1_ (2.5 kg palm^−1^)	T_1_ (8 Bunches)	15.78 ^AE^	0.712 ^CI^	0.608 ^AG^	771.4 ^CH^	561.26 ^BG^	0.150 ^GI^
		T_2_ (10 Bunches)	15.54 ^AE^	0.724 ^BH^	0.613 ^AG^	709.7 ^EI^	574.45 ^BF^	0.147 ^HI^
		T_3_ (12 Bunches)	17.80 ^AC^	0.731 ^BH^	0.611 ^AG^	724.4 ^DI^	588.04 ^BE^	0.145 ^HI^
	F_2_ (5 kg palm^−1^)	T_1_ (8 Bunches)	6.38 ^IJ^	0.841 ^A^	0.565 ^G^	383.0 ^I^	289.23 ^H^	0.197 ^AB^
		T_2_ (10 Bunches)	6.59 ^HJ^	0.841 ^A^	0.569 ^FG^	381.3 ^I^	288.37 ^H^	0.191 ^AD^
		T_3_ (12 Bunches)	5.84 ^J^	0.784 ^AD^	0.569 ^FG^	399.4 ^I^	304.01 ^GH^	0.192 ^AC^
	F_3_ (7.5 kg palm^−1^)	T_1_ (8 Bunches)	7.02 ^GJ^	0.727 ^BH^	0.602 ^BG^	408.6 ^I^	336.14 ^EH^	0.206 ^AB^
		T_2_ (10 Bunches)	7.80 ^FI^	0.777 ^AD^	0.601 ^BG^	405.0 ^I^	327.06 ^EH^	0.190 ^AD^
		T_3_ (12 Bunches)	9.85 ^EJ^	0.712 ^CI^	0.606 ^AG^	403.7 ^I^	357.46 ^EH^	0.190 ^AD^
I_3_ (120 ETc)	F_1_ (2.5 kg palm^−1^)	T_1_ (8 Bunches)	12.47 ^BI^	0.767 ^AE^	0.630 ^AF^	944.4 ^AF^	545.82 ^BH^	0.151 ^GI^
		T_2_ (10 Bunches)	12.72 ^BH^	0.708 ^DI^	0.602 ^BG^	935.6 ^AF^	486.60 ^CH^	0.176 ^BG^
		T_3_ (12 Bunches)	15.59 ^AE^	0.732 ^BH^	0.608 ^AG^	912.3 ^BF^	574.45 ^BF^	0.151 ^GI^
	F_2_ (5 kg palm^−1^)	T_1_ (8 Bunches)	6.29 ^IJ^	0.731 ^BH^	0.591 ^DG^	422.6 ^I^	316.68 ^FH^	0.201 ^AB^
		T_2_ (10 Bunches)	6.32 ^IJ^	0.766 ^AE^	0.571 ^FG^	419.7 ^I^	322.25 ^FH^	0.202 ^AB^
		T_3_ (12 Bunches)	6.35 ^IJ^	0.732 ^BH^	0.582 ^EG^	411.8 ^I^	326.47 ^EH^	0.203 ^A^
	F_3_ (7.5 kg palm^−1^)	T_1_ (8 Bunches)	9.85 ^EJ^	0.727 ^BH^	0.608 ^AG^	616.1 ^FI^	334.06 ^EH^	0.159 ^EH^
		T_2_ (10 Bunches)	6.94 ^GJ^	0.710 ^CI^	0.606 ^AG^	452.0 ^HI^	335.06 ^EH^	0.150 ^GI^
		T_3_ (12 Bunches)	6.51 ^IJ^	0.753 ^BG^	0.606 ^AG^	437.0 ^HI^	355.67 ^FH^	0.149 ^GI^
I_4_ (140% ETc)	F_1_ (2.5 kg palm^−1^)	T_1_ (8 Bunches)	12.72 ^BH^	0.775 ^AD^	0.632 ^AE^	777.3 ^CH^	523.34 ^BH^	0.176 ^BG^
		T_2_ (10 Bunches)	15.54 ^AE^	0.780 ^AD^	0.637 ^AE^	882. 5 ^BF^	523.34 ^BH^	0.176 ^BG^
		T_3_ (12 Bunches)	15.78 ^AE^	0.775 ^AD^	0.632 ^AF^	834. 7 ^BG^	523.95 ^BH^	0.180 ^AF^
	F_2_ (5 kg palm^−1^)	T_1_ (8 Bunches)	6.31 ^IJ^	0.788 ^AB^	0.592 ^CG^	460.8 ^HI^	407.17 ^DH^	0.165 ^CH^
		T_2_ (10 Bunches)	6.51 ^IJ^	0.770 ^AD^	0.598 ^BG^	455. 0 ^HI^	411.80 ^I^	0.153 ^FI^
		T_3_ (12 Bunches)	6.37 ^IJ^	0.757 ^BF^	0.593 ^CG^	536.4 ^GI^	491.96 ^CH^	0.164 ^DH^
	F_3_ (7.5 kg palm^−1^)	T_1_ (8 Bunches)	8.08 ^GJ^	0.759 ^BF^	0.613 ^AG^	616.1 ^FI^	666.25 ^AD^	0.182 ^AE^
		T_2_ (10 Bunches)	8.82 ^FJ^	0.747 ^BH^	0.611 ^AG^	771.4 ^CH^	668.13 ^AD^	0.175 ^BG^
		T_3_ (12 Bunches)	12.91 ^BG^	0.740 ^BH^	0.629 ^AG^	882.5 ^BF^	695.02 ^AC^	0.183 ^AE^

Different letters in a row show significant differences referring to treatment means. The ANOVA was constructed using a three factorial completely randomized block design, i.e., four irrigation levels (I_1_, I_2_, I_3_, and I_4_), three SOP fertilizer doses (F_1_, F_2_, and F_3_), and three bunch thinning levels (T_1_, T_2_, and T_3_). The significant data were subjected to a least significant difference (LSD) test for mean comparison at 5% level of probability (*p <* 0.05).

**Table 5:** Effects of irrigation levels, SOP fertilizer doses, and bunch thinning levels on the color parameters; lightness (L), chroma (C), hue angle (h°), and color difference (∆E) of date palm cv. Sukary. Each mean data represents the averages from two seasons, 2020/2021–2021/2022.

Irrigation Levels	SOP Fertilizer Doses	Thinning Levels (Bunches Palm^−1^)	*L*	*C*	*h*°	∆*E*
I_1_ (80% ETc)	F_1_ (2.5 kg palm^−1^)	T_1_ (8 Bunches)	42.33 ^A^	40.80 ^AB^	70.30 ^A^	58.79 ^A^
		T_2_ (10 Bunches)	44.42 ^A^	40.99 ^A^	70.38 ^A^	60.44 ^A^
		T_3_ (12 Bunches)	45.68 ^A^	41.03 ^A^	70.50 ^A^	61.40 ^A^
	F_2_ (5 kg palm^−1^)	T_1_ (8 Bunches)	29.50 ^EJ^	39.83 ^AC^	60.14 ^DH^	49.56 ^AC^
		T_2_ (10 Bunches)	30.24 ^EI^	39.18 ^AC^	61.81 ^CG^	49.49 ^AC^
		T_3_ (12 Bunches)	31.23 ^EH^	38.98 ^BC^	62.07 ^AG^	49.95 ^AC^
	F_3_ (7.5 kg palm^−1^)	T_1_ (8 Bunches)	29.72 ^EI^	40.41 ^AB^	64.56 ^AG^	50.16 ^AB^
		T_2_ (10 Bunches)	27.18 ^FK^	41.16 ^A^	64.90 ^AG^	49.32 ^AC^
		T_3_ (12 Bunches)	29.87 ^EI^	41.33 ^A^	70.54 ^A^	51.00 ^AB^
I_2_ (100% ETc)	F_1_ (2.5 kg palm^−1^)	T_1_ (8 Bunches)	33.95 ^CF^	40.82 ^AB^	65.34 ^AE^	53.09 ^AB^
		T_2_ (10 Bunches)	32.65 ^DG^	39.69 ^AC^	64.85 ^AG^	51.40 ^AB^
		T_3_ (12 Bunches)	34.86 ^BE^	41.09 ^A^	66.83 ^AE^	53.89 ^AB^
	F_2_ (5 kg palm^−1^)	T_1_ (8 Bunches)	21.05 ^K^	41.20 ^A^	51.51 ^H^	46.27 ^C^
		T_2_ (10 Bunches)	21.15 ^K^	41.32 ^A^	52.45 ^H^	46.42 ^C^
		T_3_ (12 Bunches)	21.45 ^K^	40.83 ^AB^	52.31 ^H^	46.12 ^C^
	F_3_ (7.5 kg palm^−1^)	T_1_ (8 Bunches)	23.96 ^HK^	39.90 ^AC^	60.25 ^DH^	46.54 ^C^
		T_2_ (10 Bunches)	24.39 ^HK^	39.61 ^AC^	61.88 ^CG^	46.51 ^C^
		T_3_ (12 Bunches)	26.14 ^GK^	38.36 ^BC^	63.19 ^AG^	46.42 ^C^
I_3_ (120% ETc)	F_1_ (2.5 kg palm^−1^)	T_1_ (8 Bunches)	42.93 ^A^	39.81 ^AC^	64.99 ^AG^	58.55 ^A^
		T_2_ (10 Bunches)	39.90 ^AD^	40.75 ^AB^	67.52 ^AD^	57.03 ^A^
		T_3_ (12 Bunches)	45.74 ^A^	39.12 ^AC^	67.64 ^AD^	60.19 ^A^
	F_2_ (5 kg palm^−1^)	T_1_ (8 Bunches)	21.05 ^K^	38.79 ^BC^	57.37 ^FH^	44.14 ^D^
		T_2_ (10 Bunches)	21.16 ^K^	39.08 ^AC^	57.01 ^FH^	44.44 ^D^
		T_3_ (12 Bunches)	21.46 ^K^	38.56 ^BC^	59.05 ^EH^	44.13 ^D^
	F_3_ (7.5 kg palm^−1^)	T_1_ (8 Bunches)	24.39 ^HK^	39.83 ^AC^	63.38 ^AG^	46.71 ^C^
		T_2_ (10 Bunches)	25.01 ^HK^	41.29 ^A^	63.19 ^AG^	48.27 ^AC^
		T_3_ (12 Bunches)	25.35 ^GK^	39.81 ^AC^	64.63 ^AG^	48.13 ^AC^
I_4_ (140% ETc)	F_1_ (2.5 kg palm^−1^)	T_1_ (8 Bunches)	41.26 ^AC^	40.28 ^AB^	67.09 ^AD^	57.66 ^A^
		T_2_ (10 Bunches)	41.79 ^AB^	40.68 ^AB^	65.78 ^AE^	58.32 ^A^
		T_3_ (12 Bunches)	44.74 ^A^	40.73 ^AB^	70.58 ^A^	60.50 ^A^
	F_2_ (5 kg palm^−1^)	T_1_ (8 Bunches)	22.30 ^JK^	38.18 ^BC^	60.72 ^DH^	44.21 ^D^
		T_2_ (10 Bunches)	23.62 ^IK^	38.68 ^BC^	59.31 ^EH^	45.32 ^C^
		T_3_ (12 Bunches)	23.62 ^IK^	37.80 ^C^	61.50 ^CG^	44.57 ^D^
	F_3_ (7.5 kg palm^−1^)	T_1_ (8 Bunches)	27.97 ^EK^	39.65 ^AC^	64.75 ^AG^	48.52 ^AC^
		T_2_ (10 Bunches)	27.63 ^EK^	39.35 ^AC^	64.68 ^AG^	48.08 ^AC^
		T_3_ (12 Bunches)	29.94 ^EI^	40.96 ^A^	63.98 ^AG^	50.74 ^AB^

Different letters in a row show significant differences referring to treatment means. The ANOVA was constructed using a three factorial completely randomized block design, i.e., four irrigation levels (I_1_, I_2_, I_3_, and I_4_), three SOP fertilizer doses (F_1_, F_2_, and F_3_), and three bunch thinning levels (T_1_, T_2_, and T_3_). The significant data were subjected to a least significant difference (LSD) test for mean comparison at 5% level of probability (*p <* 0.05).

**Table 6:** Effects of irrigation levels, SOP fertilizer doses, and bunch thinning levels on fruit grading (third, second, first, and excellent), skin separated fruits, biser fruits, and total fruit yield of date palm cv. Sukary. Each mean data represents the averages from two seasons, 2020/2021–2021/2022.

Irrigation Levels	SOP Fertilizer Doses	Thinning Levels (Bunches Palm^−1^)	Third Grade (%)	Second Grade (%)	First Grade (%)	Excellent Grade (%)	Skin Separated Fruits (%)	Biser Fruit (%)	Fruit Yield (kg/Palm)
I_1_ (80% ETc)	F_1_ (2.5 kg palm^−1^)	T_1_ (8 Bunches)	38.37 ^A^	23.16 ^AB^	0.02 ^C^	0.67 ^E^	25.17 ^AC^	12.61 ^AD^	33.26 ^Q^
		T_2_ (10 Bunches)	36.01 ^A^	22.47 ^AB^	1.31 ^C^	0.52 ^E^	29.00 ^AB^	10.70 ^AD^	33.60 ^Q^
		T_3_ (12 Bunches)	38.99 ^A^	24.99 ^AB^	1. 65 ^C^	0.49 ^E^	20.15 ^DF^	13.73 ^AD^	32.08 ^R^
	F_2_ (5 kg palm^−1^)	T_1_ (8 Bunches)	26.94 ^AB^	26.41 ^AB^	1.04 ^C^	0.55 ^E^	34.00 ^A^	11.06 ^AD^	35.45 ^NO^
		T_2_ (10 Bunches)	30.68 ^AB^	25.62 ^AB^	0.94 ^C^	0.49 ^E^	31.68 ^A^	16.80 ^AD^	37.30 ^L^
		T_3_ (12 Bunches)	32.34 ^AB^	22.31 ^AB^	0.97 ^C^	0.10 ^E^	33.28 ^A^	11.00 ^AD^	35.23 ^O^
	F_3_ (7.5 kg palm^−1^)	T_1_ (8 Bunches)	16.00 ^AB^	27.31 ^AB^	0.83 ^C^	0.40 ^E^	31.49 ^A^	24.11 ^A^	39.60 ^J^
		T_2_ (10 Bunches)	13.96 ^AB^	31.09 ^A^	0.89 ^C^	0.51 ^E^	33.55 ^A^	20.00 ^AD^	39.70 ^J^
		T_3_ (12 Bunches)	18.66 ^AB^	25.34 ^AB^	0.86 ^C^	0.11 ^E^	33.10 ^A^	21.93 ^AB^	38.47 ^K^
I_2_ (100% ETc)	F_1_ (2.5 kg palm^−1^)	T_1_ (8 Bunches)	15.07 ^AB^	14.68 ^B^	37.57 ^AB^	14.53 ^D^	7.57 ^BC^	10.58 ^AD^	38.12 ^K^
		T_2_ (10 Bunches)	15.63 ^AB^	20.21 ^AB^	36.00 ^AB^	24.25 ^AC^	7.22 ^BC^	11.87 ^AD^	37.59 ^L^
		T_3_ (12 Bunches)	18.29 ^AB^	14.41 ^B^	33.55 ^AB^	15.23 ^D^	6.90 ^BC^	11.62 ^AD^	35.03 ^O^
	F_2_ (5 kg palm^−1^)	T_1_ (8 Bunches)	13.80 ^AB^	14.40 ^B^	33.00 ^AB^	19.71 ^CD^	2.26 ^C^	1.65 ^D^	62.90 ^B^
		T_2_ (10 Bunches)	12.95 ^AB^	21.89 ^AB^	30.79 ^AB^	29.49 ^A^	2.38 ^C^	2.50 ^CD^	68.00 ^A^
		T_3_ (12 Bunches)	11.26 ^AB^	21.81 ^AB^	34.52 ^AB^	20.60 ^BD^	2.99 ^C^	5.59 ^BD^	62.78 ^B^
	F_3_ (7.5 kg palm^−1^)	T_1_ (8 Bunches)	15.39 ^AB^	19.35 ^AB^	32.98 ^AB^	20.00 ^BD^	2.96 ^C^	10.82 ^AD^	59.32 ^EF^
		T_2_ (10 Bunches)	17.44 ^AB^	20.34 ^AB^	25.26 ^B^	28.69 ^AB^	2.68 ^C^	8.79 ^AD^	60.23 ^D^
		T_3_ (12 Bunches)	15.13 ^AB^	19.94 ^AB^	29.41 ^AB^	19.72 ^CD^	4.33 ^C^	12.83 ^AD^	58.12 ^GH^
I_3_ (120% ETc)	F_1_ (2.5 kg palm^−1^)	T_1_ (8 Bunches)	15.38 ^AB^	15.01 ^B^	36.34 ^AB^	14.67 ^D^	7.73 ^BC^	15.38 ^AB^	39.94 ^J^
		T_2_ (10 Bunches)	14.21 ^AB^	14.81 ^B^	35.54 ^AB^	16.04 ^CD^	7.41 ^BC^	11.99 ^AD^	38.17 ^K^
		T_3_ (12 Bunches)	19.16 ^AB^	15.12 ^B^	30.11 ^AB^	16.17 ^CD^	7.24 ^BC^	12.18 ^AD^	35.86 ^N^
	F_2_ (5 kg palm^−1^)	T_1_ (8 Bunches)	17.10 ^AB^	22.05 ^AB^	43.76 ^A^	12.82 ^D^	2.47 ^C^	1.80 ^D^	59.38 ^EF^
		T_2_ (10 Bunches)	14.22 ^AB^	24.16 ^AB^	43.31 ^A^	13.06 ^D^	2.38 ^C^	2.95 ^CD^	61.15 ^C^
		T_3_ (12 Bunches)	17.33 ^AB^	21.25 ^AB^	40.41 ^AB^	13.30 ^D^	2.14 ^C^	5.57 ^BD^	60.13 ^D^
	F_3_ (7.5 kg palm^−1^)	T_1_ (8 Bunches)	15.27 ^AB^	19.11 ^AB^	39.57 ^AB^	12.20 ^D^	2.97 ^C^	10.88 ^AD^	58.56 ^G^
		T_2_ (10 Bunches)	11.40 ^AB^	22.03 ^AB^	42.47 ^A^	12.58 ^D^	3.03 ^C^	8.94 ^AD^	57.00 ^I^
		T_3_ (12 Bunches)	16.13 ^AB^	21.25 ^AB^	33.58 ^AB^	12.19 ^D^	3.15 ^C^	13.70 ^AD^	57.66 ^H^
I_4_ (140% ETc)	F_1_ (2.5 kg palm^−1^)	T_1_ (8 Bunches)	25.99 ^AB^	25.67 ^AB^	0.99 ^C^	0.67 ^E^	35.97 ^A^	10.79 ^AD^	36.74 ^M^
		T_2_ (10 Bunches)	25.46 ^AB^	23.74 ^AB^	1.33 ^C^	0.44 ^E^	33.97 ^A^	15.06 ^AD^	35.36 ^NO^
		T_3_ (12 Bunches)	27.65 ^AB^	20.42 ^AB^	0.88 ^C^	0.49 ^E^	35.45 ^A^	15.12 ^AD^	34.41 ^P^
	F_2_ (5 kg palm^−1^)	T_1_ (8 Bunches)	28.76 ^AB^	26.36 ^AB^	1.03 ^C^	1.01 ^E^	32.12 ^A^	10.72 ^AD^	59.22 ^F^
		T_2_ (10 Bunches)	31.74 ^AB^	22.00 ^AB^	1.26 ^KC^	0.44 ^E^	31.87 ^A^	12.69 ^AD^	59.73 ^DE^
		T_3_ (12 Bunches)	33.20 ^AB^	23.20 ^AB^	1.22 ^C^	0.91 ^E^	33.20 ^A^	18.37 ^AD^	58.50 ^G^
	F_3_ (7.5 kg palm^−1^)	T_1_ (8 Bunches)	16.91 ^AB^	25.82 ^AB^	1.01 ^C^	0.49 ^E^	36.75 ^A^	19.01 ^AD^	57.71 ^H^
		T_2_ (10 Bunches)	18.49 ^AB^	26.20 ^AB^	1.45 ^C^	0.50 ^E^	37.25 ^A^	16.09 ^AD^	58.15 ^GH^
		T_3_ (12 Bunches)	18.99 ^AB^	26.30 ^AB^	1.03 ^C^	0.53 ^E^	37.03 ^A^	24.06 ^AD^	56.56 ^I^

Different letters in a row show significant differences referring to treatment means. The ANOVA was constructed using a three factorial completely randomized block design, i.e., four irrigation levels (I_1_, I_2_, I_3_, and I_4_), three SOP fertilizer doses (F_1_, F_2_, and F_3_), and three bunch thinning levels (T_1_, T_2_, and T_3_). The significant data were subjected to a least significant difference (LSD) test for mean comparison at 5% level of probability (*p <* 0.05).

**Table 7:** Average monthly values of the temperature (Temp), relative humidity (RH), wind speed (WS), solar radiation (S. Rad), and air pressure (AP) of Al-Qassim region, KSA, during the seasons 2020/2021–2021/2022.

Months	Season 2020/2021					Season 2021/2022				
	Temp (°C)	RH (%)	WS (km)	S. Rad (W m^−2^)	AP (psi)	Temp (°C)	RH (%)	WS (km)	S. Rad (W m^−2^)	AP (psi)
JAN	16.77	45.15	2.61	173.07	14.78	16.42	51.07	2.87	175.68	14.76
FEB	18.93	42.63	2.55	213.55	14.75	18.15	45.47	3.15	221.41	14.73
MAR	24.05	25.55	3.16	231.63	14.69	23.34	25.98	3.54	238.11	14.71
APR	29.68	18.99	3.01	243.80	14.66	29.17	19.47	3.41	249.88	14.65
MAY	35.26	14.59	2.86	289.89	14.57	33.54	14.62	3.34	297.57	14.61
JUN	38.70	11.42	2.92	356.29	14.53	38.76	12.17	3.42	341.04	14.52
JUL	39.13	11.03	3.10	314.19	14.47	38.89	12.85	3.19	322.08	14.47
AUG	38.65	12.40	3.49	295.18	14.52	38.71	12.74	3.21	301.47	14.49
SEP	34.52	11.88	2.59	277.30	14.56	35.55	13.47	2.38	281.63	14.57
OCT	30.51	22.65	2.63	241.50	14.66	30.49	24.98	2.74	249.85	14.68
NOV	24.72	37.22	2.93	174.24	14.72	25.17	37.84	2.99	175.84	14.72
DEC	18.44	42.43	2.30	170.85	14.76	19.09	52.47	2.44	171.91	14.72

## Data Availability

Not applicable.
